# Positively charged liposomal β-carotene enhances irradiation-induced cytotoxicity against prostate carcinoma cells: an in-vitro study

**DOI:** 10.1186/s12885-025-15244-z

**Published:** 2025-12-10

**Authors:** Mahmoud Abdelrahman, Medhat W. Shafaa, Hany Abdelrahman, Moustafa Ibrahim

**Affiliations:** 1 Physics Department, Faculty of Science, Benha University, Benha, Qalyubia Egypt; 2 Medical Biophysics Division, Physics Department, Faculty of Science, Helwan University, Cairo, Egypt; 3 Departments of Nuclear Medicine, Adan Hospital, Kuwait, Al-Adan Kuwait

**Keywords:** Β-Carotene, Cationic liposomes, FTIR, DSC, Prostate cancer (PC-3), Gamma irradiation

## Abstract

Interactions of β-carotene in the absence or presence of stearylamine (SA, positive charge inducer) with liposomes as model membranes were characterized. The morphology of all liposomes was almost spherical in shape, well dispersed and less aggregated for empty and encapsulated vesicles. The mean particle size distribution of empty liposomal samples was clustered around 43.82 ± 21.29 nm. The presence of SA in liposomal membranes decreased the density of negative charge, resulting in a positive zeta potential. The introduction of β-carotene or β-carotene mixed with SA into liposomes exhibited a shift to higher temperature. FTIR study confirmed the interaction of β-carotene or β-carotene mixed with SA with the liposome’s moieties. In the absence of external gamma irradiation, the IC₅₀ value for free β-carotene in PC-3 cells was 29.24 µg/ml, whereas positively charged liposomal β-carotene showed an IC₅₀ of 34.74 µg/ml, indicating slightly greater cytotoxicity of the free form when used alone. However, under combined treatment with 5 Gy gamma irradiation, the IC₅₀ for positively charged liposomal β-carotene was markedly reduced to 11.66 µg/ml compared with 32.46 µg/ml for free β-carotene, demonstrating the superior cytotoxic effect of the charged liposomal formulation in the presence of irradiation. Flow cytometric analysis revealed that after 48 h of exposure to positively charged liposomal β-carotene. The treatment with the IC50 of positively charged liposomal β-carotene resulted in (58.14% and 33.67%) followed by liposomal β-carotene (51.52% and 43.66%) cell accumulation in the G0-G1 and S phases, respectively. The current findings demonstrated that natural product preparations could be a viable therapy option for prostate cancer in place of pharmaceutical therapies.

## Introduction

 Cancer is a term used to describe a group of diseases in which abnormal cells develop and spread uncontrollably. If the tumor’s prevalence is not monitored, it can lead to death. It is the most common secondary cause of death, with only heart disease accounting for one out of every four deaths. Surgical, radiotherapy, chemotherapy, hormonal therapy, immunological therapy, and targeted therapy are among of the options for treatment [1].

The incidence of Prostate Cancer has been on the rise in recent years, and there is a need to explore different treatment modalities that may influence the growth of different types of cancer cells and their consequent therapeutic activity [2]. In particular, the focus has been on dietary factors that may have an impact on the risk of prostate cancer, including carotenoids and other chemo-preventive compounds. In such a concept, the use of antioxidants in liposomes has emerged as a promising strategy to alter the physicochemical properties of liposomes and their therapeutic activity. Such a strategy encompasses increasing the stability of β-carotene towards oxidative breakdown and raising its permeability when utilized as a delivering system.

Apoptosis is used by common cancer therapies like chemotherapy to eradicate dangerous cells from tumors [3, 4]. Although this approach is effective against a wide range of tumor types, it is not very selective and adversely affects healthy tissue. A more effective way to treat cancer would be to selectively discriminate against malignant cells from normal cells using tiny molecules [5].

Nanocarriers used to cure cancer have made great progress in lipid-based nanocarriers. Different forms of lipid formulations are currently available, such as lipid nanoparticles or nanostructured lipid carriers. Because of their bio compliance and biodegradability, these lipid-based models are less toxic than other drug delivery systems, such as Polymeric nanoparticles. Liposomes are spherical vehicles artificially generated with a lipid bilayer surrounding a hollow center, which can be filled with chemotherapeutic drugs for delivery to tumor sites [6, 7]. According to its effectiveness, biocompatibility, non-immunogenicity, enhanced solubility of chemical agents, and their ability to encapsulate a broad range of drugs, these nano-sized lipid bilayer vesicles have become common for drug delivery systems. Liposomal delivery of anticancer agents enhances their therapeutic potential in part by reducing exposure to healthy tissues or by enabling lower drug doses to achieve effective tumor cell toxicity. The interaction between liposomes and cells is largely influenced by the surface charge and its distribution. Modifying the lipid composition of liposomes can adjust both their charge and their interaction behavior. Negatively charged liposomes tend to have stronger interactions with cells, which facilitates greater cellular uptake. In contrast, positively charged (cationic) liposomes, although capable of delivering their payload through membrane fusion, are typically cleared from circulation more quickly following systemic administration. Lecithin is the name given to a mixture of glycolipids, triglycerides, and phospholipids (for example phosphatidylcholine, phosphatidylethanolamine, and phosphatidylinositol). However, in biochemistry, the term lecithin is usually used as a synonym for pure phosphatidylcholine - a phospholipid which is the main component of the phosphate fraction obtained from egg yolk. Lecithin is considered as a non-toxic surfactant which is well tolerated by the organism, since it is an integral part of cell membranes and can be completely metabolized. Lecithin can be used for producing liposomal structures due to its safety and suitable accessibility [8, 9].

Liposomes were selected in the present study as the delivery system for β-carotene because of their biocompatibility, ability to encapsulate hydrophobic compounds, and proven role in enhancing stability against oxidative degradation. Encapsulation of carotenoids into liposomes not only improves their solubility and bioavailability but also shields them from degradation during storage and under physiological conditions, thereby maintaining their therapeutic activity. In particular, positively charged liposomes, prepared by incorporating stearylamine (SA) as a charge inducer, were utilized in order to enhance colloidal stability by reducing vesicle aggregation and to facilitate stronger electrostatic interactions with negatively charged cellular membranes. Such interactions are expected to improve cellular uptake and thereby potentiate the cytotoxic activity of encapsulated β-carotene. This dual approach—liposomal encapsulation and surface charge modification—offers a rational strategy for developing more stable and effective nanocarriers of natural antioxidants such as β-carotene for cancer therapy.

Radiation therapy is a treatment method for cancer that is commonly used when the tumor is limited to a specific region of the body that can be physically targeted. Because it destroys the DNA or genes that control cell replication and development, radiation therapy is effective as a cancer treatment. By directly absorbing radiation into DNA, radiation can cause DNA damage and cell death, or it can trigger the creation of free radicals by ionizing intracellular water molecules, particularly those in the DNA environment [10–12].

A combination of anticancer drug and ionized radiation therapy is a popular treatment for various cancers. During therapy, these two agents have significant synergy, but the synergy mechanism is largely unknown. The mechanism for radio-therapeutic sensitization with cisplatin has been investigated in vitro breast cancer, which continues to be a significant global health burden. Since the majority of tumors are diagnosed locally, radiation is the most effective treatment option.

Carotenoids represent a class of lipophilic pigments with a polyisoprenoid structure derived from plants. Epidemiological investigations have revealed a correlation between higher circulating levels of dietary carotenoids, such as α-carotene, β-carotene, lycopene, and lutein/zeaxanthin, and a reduced risk of cancer [13].

Epidemiological research has revealed an inverse correlation between cancer risk and the consumption of green and yellow vegetables, along with fruits. Given that β-carotene is abundantly present in these plant-based foods, it has been extensively investigated as a potential cancer-preventive agent. Numerous studies have underscored the anti-carcinogenic activity of β-carotene found in our daily dietary intake [14–16].

Furthermore, previous studies have illuminated the diverse biological properties of β-carotene, encompassing anti-inflammatory, antioxidant, and anticancer effects. Its capacity for growth inhibition and cytotoxicity has been demonstrated across various cancer cell lines and animal models, further solidifying its potential as a valuable component in cancer prevention and treatment strategies [17].

Natural treatment could be the best replacement for the treatment of cancer, avoiding the various physical side effects caused by chemotherapy and radiation therapy. This treatment can effectively avoid the damaging of normal, healthy cells near cancer cells [10, 12].

To date, the anti-proliferative activity of β-carotene in free and Nano liposomal forms towards PC-3 (prostate cancer cell line) in the absence or presence of external gamma-irradiation has not yet been studied.

This study was undertaken to examine how β-carotene in the absence or presence of Stearylamine (SA, positive charge inducer) modifies the properties of the physical structure of the lecithin as a lipidic model membrane by the estimation of the accurate disruption of the bilayer lipid structure using FTIR (Fourier transform infrared), transmission electron microscopy (TEM), Dynamic Light Scattering (DLS), Polydispersity (PDI), Zeta Potential, and DSC (Differential Calorimetry scanning). The study also examines the cytotoxic effectiveness of free β-carotene or its formulation into liposomes by discovering the prospective effect of all of these tested compounds on the in vitro survival of PC-3 (prostate cancer cell line) following combined treatment with gamma radiation at 5 Gy dose.

## Materials and methods

### Chemicals

**Fig. 1:**
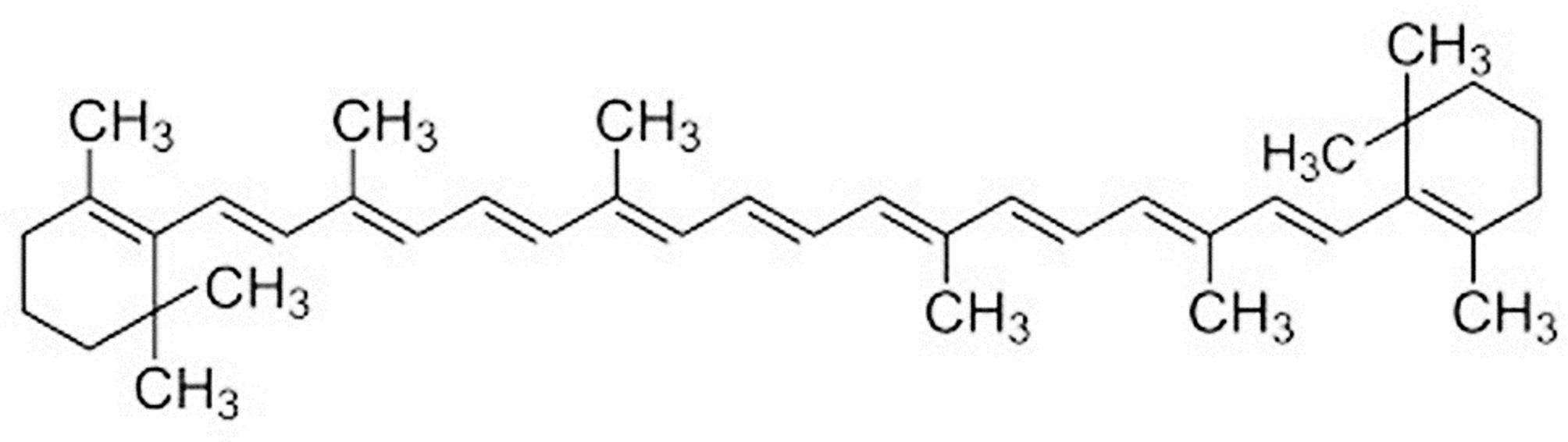
The chemical structure of β-carotene

β-carotene was meticulously extracted and purified from natural sources, undergoing thorough verification of purity through spectral analysis. Spectral data confirmed the molecular structure of β-carotene, with a molecular weight of 536.9, as depicted in Fig. 1.

**Fig. 2:**
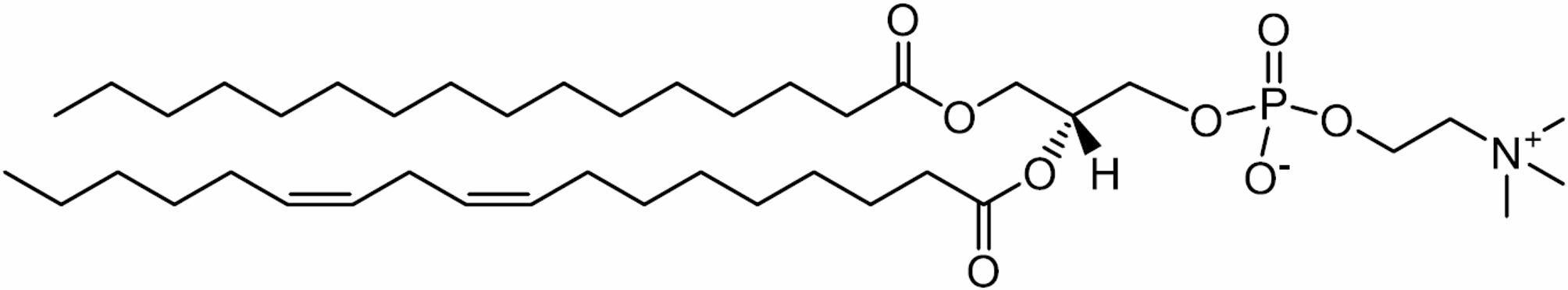
Schematic chemical structure of L-α-phosphatidyl choline (Soy Lecithin)

Ethanol alcohol, with an absolute 99.9%, was bought from DaeJung Chemicals (Seohaean-ro, Gyeonggi-do, Korea). L-α-phosphatidyl choline (Soy Lecithin) was purchased from Carl Roth (Karlsruhe, Germany) with a molecular weight of 760 in powder form and a purity of ≥ 97% (Fig. 2). Phosphate buffer saline was sourced from CDH, New Delhi, India. PC-3 (Human Prostate cancer cell line) was put in a −180 ° C (liquid nitrogen) according to “The American Form Culture Array”. Then it was kept by serial sub-culturing in “Vacsera Vaccination Center, Cairo, Egypt”. Dimethylsulphoxide (DMSO), DMEM medium, Sodium bicarbonate, Trypan blue (0.05% isotonic solution in normal saline), Penicillin/Streptomycin, Trypsin, Acetic acid, Fetal bovine serum (FBS), Sulphorhodamine-B (SRB), 3-(4,5-dimethylthiazol-2-yl) 2,5 diphenyl tetrazolium bromide (MTT), Poly ethylene Glycol (PEG) and Trichloroacetic acid (TCA) were obtained from Sigma Chemical Co., St. Louis, Mo, USA. Additionally, 0.4% SRB dissolved in 1% acetic acid and 100% isopropanol were procured for experimentation. All solutions were prepared using distilled ultra-pure water, and all solvents and reagents were of HPLC grade.

Preparation of liposomes: Neutral multilamellar vesicles (MLVs) were formulated using soy lecithin and β-carotene in a molar ratio of 7:2, employing the thin-film hydration technique originally described by Bangham [18]. Specifically, 100 mg of soy lecithin and 20 mg of β-carotene were placed in a 100 ml round-bottom flask, followed by the addition of 25 ml of ethanol (EtOH). The mixture was agitated until complete dissolution of both components. The ethanol was then slowly evaporated under reduced pressure using a rotary evaporator in a 55 °C water bath, forming a consistent lipid film along the inner surface of the flask. This film was hydrated with phosphate-buffered saline (PBS, pH 7.4 at 37 °C) for 15 min at 55 °C, leading to the formation of MLVs. The hydration process was enhanced by mechanical shaking of the flask at 55 °C for 1 h, followed by bath sonication to decrease the vesicle size and promote the formation of Unilamellar vesicles. Afterward, the flask was purged with nitrogen and immediately sealed. A control formulation of empty liposomes was prepared using the same protocol but without β-carotene, containing only the measured soy lecithin. To impart a net positive surface charge, aliquots of stearylamine (SA) were incorporated into the lipid mixture during preparation.

### Encapsulation efficiency measurement

Encapsulation efficiency was measured through the method proposed by Shafaa et al. (2007). It is a measure of carotenoid extraction from liposomal suspensions, then quantified through UV–Vis absorption spectrophotometry. It is a means through which the incorporation yield is calculated in the form of a ratio of the effective concentration (EC) to incubation concentration (IC) [19]. To obtain the free amount of β-carotene in suspension, 2 mL of ethanol and aliquots of 100 µl of β-carotene -loaded liposomes were firmly combined for 3 min at ambient temperature by vortexing. For 20 min at 6000 rpm ×g., the mixed sample was centrifuged to remove the supernatant from the centrifuged sample. The β-carotene absorbance was measured at around 450 nm by using a UV-Vis spectrophotometer (Jasco V-630, Germany). Using the following formula to calculate the β-carotene extracts, known volumes of β-carotene solution were used to evaluate the β-carotene weight by measurement. As a triplet, each experiment was performed.

$$\:W\left(mg\right)=\frac{A*V*{10}^{3}}{{A}_{1cm}^{1\%}*100}*DF$$

Where, W denotes the carotenoid weight and A denotes the optical density (which should be between 0.2 and 0.8). The volume of the β-carotene -containing solution is V., $\:{A}_{1cm}^{1\%}$ is the specific extinction coefficient of each carotenoid in the solvent used [2550 for β-carotene in ethanol] and $\:DF$ is the dilution factor [20].

### Determination of entrapment efficiency percentage

The entrapment efficiency (EE%) was calculated through the following relationship:

1 $$\begin{aligned} \:EE\%=&;\frac{Total\:drug\:input\:\left(\text{mg}\right)-Drug\:in\:supernatant\left(\text{mg}\right)}{Total\:drug\:input\left(\text{mg}\right)}\\&;\times\:100 \end{aligned}$$

Where Total drug input refers to the initial mass of β-carotene added during liposome preparation, and free drug in supernatant represents the un-encapsulated β-carotene quantified from the supernatant after centrifugation.

### Liposome morphology by transmission electron microscopy

The structural characteristics and morphology of the liposomal formulations - including blank liposomes, β-carotene-loaded liposomes, and positively charged β-carotene liposomes - were examined using transmission electron microscopy (TEM). Imaging was conducted on a JEOL JEM-2100 transmission electron microscope (Japan) operating at an accelerating voltage of 200 kV. For contrast enhancement, samples were negatively stained with a 1% (w/v) aqueous solution of phosphotungstic acid. Prior to imaging, liposome samples were diluted at a ratio of 1:10 in Tris buffer (pH 7.4, 37 °C). A 20 µL aliquot of each sample was applied to a carbon-coated copper TEM grid and allowed to adsorb for 1 min. Excess liquid was gently removed using filter paper. The resulting grids were then examined under TEM, and representative micrographs were acquired for analysis.

### Dynamic light scattering and zeta potential analysis

The average particle size, polydispersity index (PDI), and zeta potential of the prepared formulations-blank liposomes, β-carotene-loaded liposomes, and positively charged β-carotene liposomes-were evaluated using dynamic light scattering (DLS). Measurements were performed using a particle sizing system (PSS, Santa Barbara, CA, USA) in Tris buffer (pH 7.4) at 25 °C. Each formulation was analyzed in triplicate, and results are reported as mean values ± standard deviation.

### Differential Scanning Calorimetry (DSC)

The thermal properties of lyophilized liposomal formulations, including blank liposomes, β-carotene-loaded liposomes, and positively charged liposomal β-carotene, were assessed using a differential scanning calorimeter (DSC; Setaram Labsys, France) calibrated with indium. Approximately 5 mg of each sample was hermetically sealed in standard aluminum pans. Thermal scans were conducted at a heating rate of 3 °C/min across a temperature range of 25 °C to 200 °C, and thermograms were recorded for each sample.

### Fourier-Transform Infrared (FTIR) spectroscopy

Fourier-transform infrared (FTIR) spectroscopy was employed to characterize the molecular interactions within the lyophilized liposomal formulations. Samples of blank liposomes, β-carotene-loaded liposomes, and positively charged β-carotene liposomes were compressed into potassium bromide (KBr) discs and analyzed using an Alpha II FTIR spectrometer (Bruker, Switzerland). Scanning was conducted at room temperature over the wavenumber range of 400 to 4000 cm⁻¹, with a scan speed of 2 mm/s.

### In-vitro cytotoxicity by MTT assay

The PC-3 human prostate cancer cell line was washed with phosphate-buffered saline (PBS), trypsinized, and resuspended in complete RPMI-1640 medium. Cell viability and concentration were assessed using the Trypan Blue exclusion method with a hemocytometer, as previously described [21].

For chemo-sensitivity evaluation, cells were seeded into 96-well micro-plates at a density of 1 × 10⁴ cells per well and allowed to adhere for 24 h. After incubation, the medium was aspirated and replaced with fresh RPMI-1640 containing serial two-fold dilutions of the test compounds. The cytotoxic effects of free β-carotene, blank liposomes, β-carotene-loaded liposomes, and positively charged β-carotene liposomes were individually assessed. Each concentration was tested in triplicate wells.

After 48 h of incubation at 37 °C in a humidified atmosphere containing 5% CO₂, the drug-containing medium was removed, and the cells were gently washed twice with PBS. A 5 mg/mL stock solution of MTT (3-(4,5-dimethylthiazol-2-yl)−2,5-diphenyl tetrazolium bromide) was prepared, from which 50 µL was added to each well to achieve a final working concentration of 0.5 mg/mL. The plates were incubated for an additional 4 h at 37 °C to allow for formazan crystal formation. Thereafter, the MTT solution was carefully removed, and 50 µL of dimethyl sulfoxide (DMSO) was added to dissolve the formazan crystals.

Cell viability was assessed through absorbance measurement at 490 nm in a microplate reader (Boster Immunoleader, USA). Percentage of viable cells was determined by normalizing absorbance of the drug-treated wells relative to nontreated control wells, set as 100% viability. Dose–response curves were made through plotting the viability of cells against drug concentration, and the IC₅₀ values were obtained from nonlinear regression analysis in a four-parameter logistic model.

### Single cell gel electrophoresis (comet assay)

The comet assay, a cost-effective and sensitive technique, was employed to detect various forms of DNA damage, including single- and double-strand breaks, DNA adducts, cross-links, and alkali-labile sites. The procedure was carried out based on the method described by N. P. Singh et al. [22], with modifications according to J. Blasiak et al. [23].

Microgel slides were prepared in three sequential layers. First, 100 µL of 0.7% normal melting point agarose was spread onto pre-cleaned, fully frosted microscope slides and gently covered with a coverslip. After solidification at 4 °C, the coverslip was removed. Subsequently, 0.5% low melting-point agarose prepared in 100 mmol/L phosphate-buffered saline (PBS) and maintained at 37 °C was mixed with freshly isolated mononuclear cells. A 100 µL aliquot of this cell-agarose suspension was layered onto the solidified base and covered with a coverslip. After solidification at 4 °C, the coverslip was removed, and a final layer of 0.5% low melting-point agarose was added. This final layer was also allowed to solidify at 4 °C under a coverslip, which was then removed after 10 min.

The slides were then immersed in freshly prepared lysis buffer (pH 10) containing 2.5 mol/L NaCl, 100 mmol/L EDTA, 1% sodium hydroxide, 10 mmol/L Tris, 1% Triton X-100, and 10% DMSO for 1 h at 4 °C. Following lysis, the slides were incubated in a DNA unwinding solution (300 mmol/L NaOH, 1 mmol/L EDTA, pH 13) for 30 min at 4 °C. Electrophoresis was then performed in the same solution using a horizontal electrophoresis chamber under constant current (300 mA) for 30 min at 4 °C.

After electrophoresis, the slides were neutralized with 0.4 mol/L Tris base (pH 7.5) for 10 min. DNA was visualized by staining with ethidium bromide (20 µL of a 10 µg/mL solution).

Comet images were captured using a fluorescence microscope (IX70; Olympus, Tokyo, Japan) equipped with a 549 nm excitation filter and a 590 nm barrier filter, at 400× magnification, and connected to a digital video camera. Damaged cells were identified by the characteristic “comet” appearance—comprising a bright head and a trailing tail of fragmented DNA. DNA damage was quantified by counting the number of damaged cells per 100 cells per slide, expressed as a percentage.

### Detection of apoptosis by flow cytometry

The half-maximal inhibitory concentrations (IC₅₀) of the three tested formulations—free β-carotene, blank liposomes, and β-carotene-loaded liposomes—were determined using the MTT assay and subsequently used to treat PC-3 prostate cancer cells. Cells were seeded at a density of 1 × 10⁶ cells per 25 cm² culture flask and incubated with the respective treatments for 48 h. Post-treatment, cells were harvested by trypsinization and collected via centrifugation at 2000 rpm for 10 min. The resulting cell pellets were washed with ice-cold phosphate-buffered saline (PBS) and centrifuged again at 500 ×g for 5 min at 4 °C. The supernatant was removed, and the cell pellets were resuspended in 100 µL of binding buffer containing 1 µL Annexin V-FITC and 5 µL propidium iodide (PI). Staining was carried out at room temperature in the dark for 15 min.

Following staining, 400 µL of binding buffer was added to each sample prior to analysis using a BD FACSCalibur™ flow cytometer. The extent of apoptosis was quantified and reported as apoptotic profiles based on Annexin V/PI staining.

### Cell cycle analysis

To assess cell cycle distribution, PC-3 cells were treated with IC₅₀ concentrations of free β-carotene, blank liposomes, β-carotene-loaded liposomes, and positively charged β-carotene liposomes. After 48 h of treatment, cells were fixed in 70% ethanol and stored overnight at 4 °C. The following day, fixed cells were incubated in phosphate-buffered saline (PBS) containing RNase A at a final concentration of 1.5 µg/mL for 1 h at 37 °C to degrade RNA. Subsequently, cells were stained with 5 µL of propidium iodide (PI) and incubated on ice for 20 min.

Prior to analysis, cell suspensions were filtered through a 40 μm nylon mesh to remove clumps. The DNA content of the stained cells was then analyzed using a BD FACSCalibur™ flow cytometer. Cell cycle phase distribution was evaluated and expressed as DNA content profiles.

### In vitro irradiation of PC-3 prostate cancer cell line

The PC-3 Prostate cancer cell line was first grown for 24 h in 25 cm^2^ culture flasks at a cell density of 1 × 10^4^, then treated with both the IC_50_ concentration (obtained from cytotoxicity MTT test) of the tested chemotherapeutic agents, then irradiated with gamma-ray dose of 5 Gy using a Cs^137^ unit station (0.653 rad/sec dose rate) (Gamma-cell 40, Canada).

## Results and discussion

The entrapment efficiency percentage was found to be higher than 90% for all prepared liposomal suspensions when the drug was mixed with the lipid powder before dissolving it in ethanol.

**Fig. 3:**
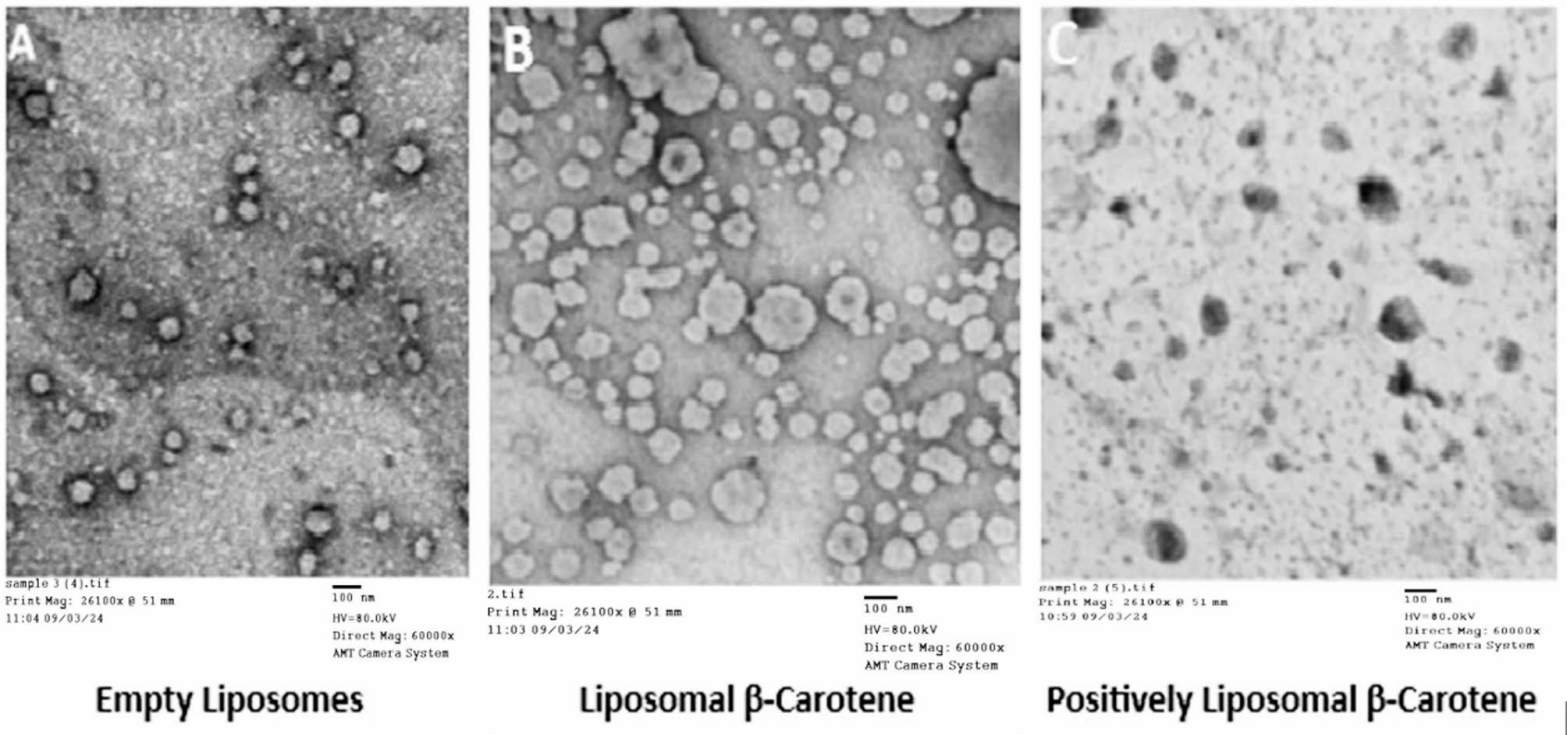
TEM images for empty liposomes (**A**), β-carotene-loaded liposomes (**B**) and positively charged liposomal β-carotene (**C**). The scale bar in all panels is 100 nm

TEM images showed that, as shown in Fig. 3, the morphology of all liposomes prepared in this work was almost spherical in shape, well dispersed and less aggregated for empty (Fig. 3A) and encapsulated β-carotene vesicles (Fig. 3A and B). TEM findings showed that β-carotene in the presence of Stearylamine (SA, positive charge inducer) can be physically correlated with disrupting the membrane packing property of liposomes on the surface (Fig. 3C). The presence of β-carotene in liposomes coated with SA (Fig. 3C) may favor or suggest enhanced interactions with the lipid bilayer of liposomes, possibly mediated by hydrogen bonding. It is supported that β-carotene could be inserted in the hydrophobic region of the bilayer and these findings are in good agreement with the data observed by the DSC and FTIR results.

Colloidal suspension particle homogeneity is effectively accounted for by the polydispersity index (PDI). Values above 0.7 mean that the sample has a very wide size range and that the dynamic light scattering technique is therefore not stable [24].

**Fig. 4:**
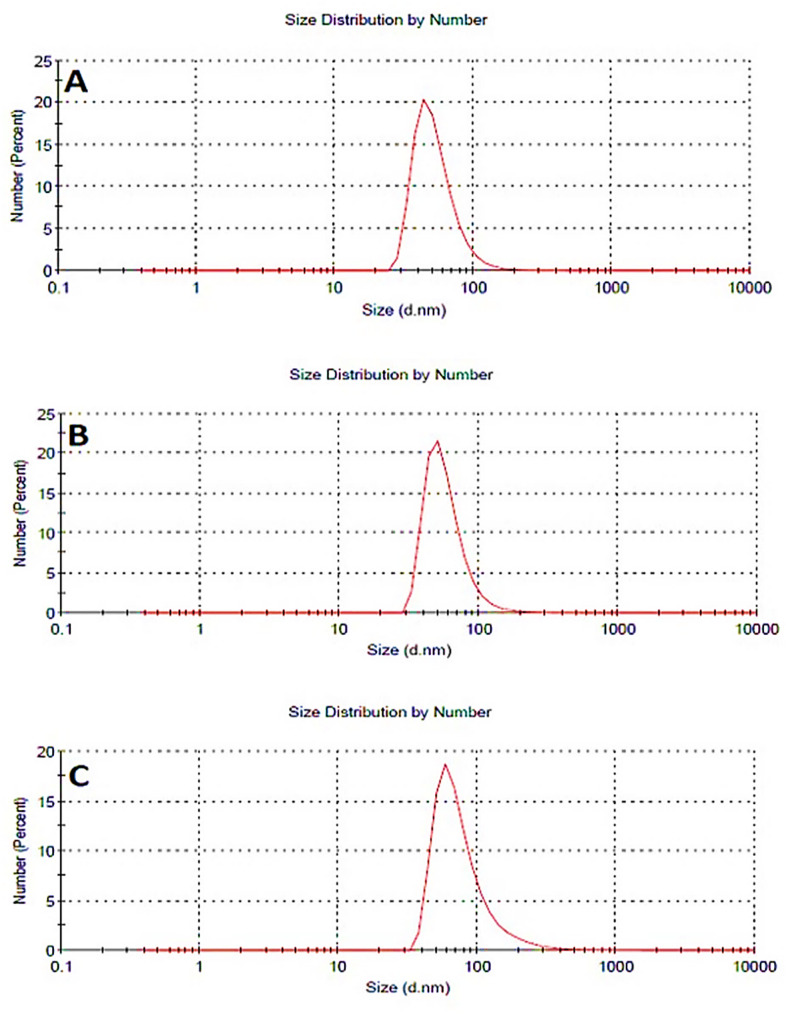
Liposomes size distribution measured by dynamic light scattering (DLS) for (**A**) empty liposomal sample, β-carotene-loaded liposomes (**B**) and positively charged liposomal β-carotene (**C**)

The sample of lecithin showed an average diameter of 43.82 ± 21.29 nm with a polydispersity index (PDI) of 0.221, as summarized in Table 1. Blank liposome size increased upon the addition of β-carotene to 50.75 ± 28.54 nm with a decreasing PDI of 0.175, indicating a more homogenous population. Growth in the size of the liposomes after the addition of β-carotene can be rationalized on the basis of increased space occurring in adjacent bilayers in the presence of the lipophilic carotenoid. Unlike customary lipid-based Nano vesicles, which normally require a co-surfactant for the vesicular wall’s stability, our formulation did not involve the use of an additional co-surfactant. Instead, stearylamine (SA) was employed, and it might serve a dual role: (i) acting as a positive charge introducer for increased colloidal stability, and (ii) acting as a co-surfactant for membrane rigidity and stabilization. Stronger β-carotene interactions with the polar head group of phospholipids via hydrogen bonding near the PO_2_^–^ group could explain the increase in particle size (Fig. 4B). The random distribution of the nonpolar β-carotene in the lipid bilayer, without any preferred orientation, results in increased motional freedom of the alkyl hydrocarbon chains in the liquid crystalline state and of the polar lipid head groups, thus fluidizing the entire lipid bilayer [25]. These results indicate that the liposomes may be physically associated with β-carotene at the core and the molecule of it tends to interact to large extent with the lipid bilayer and perturb them. The incorporation of SA into liposomal β-carotene resulted in an increase in the calculated mean size diameter of blank liposomes to 58.77 ± 22.13 nm with 0.201 PDI (Fig. 4C). When SA was added to liposomes, the spacing between neighboring bilayers increased, resulting in liposomes that were larger than control liposomes and this probably could be attributed to the electrostatic repulsive force between the soy lecithin N(CH_3_)_3_^+^ group and the SA NH_2_^+^ group within the lipid bilayer of liposomes. These findings are in good agreement with the data observed by DSC and FTIR studies.

**Table 1:** Summarized data obtained for the dynamic light scattering (DLS) and zeta potential for liposomes before and after encapsulation by β-carotene in the absence or presence of SA

Sample name	Mean size diameter (nm) ± SD (nm)	PDI average	Mean zeta potential ± SD (mV)
Empty Liposomes	43.82 ± 21.29	0.221	−31.9 ± 10.7
Liposomal β-carotene	50.75 ± 28.54	0.175	−58.8 ± 6.5
Positively charged liposomal β-carotene	58.77 ± 22.13	0.201	+ 23.9 ± 5.11

The magnitude of the zeta potential shows the colloidal system’s potential stability. As the zeta potential rises, particle repulsion increases, resulting in a more stable colloidal dispersion. If all suspended particles have a significant negative or positive zeta potential, they resist each other and are unlikely to clump together [26].

**Fig. 5:**
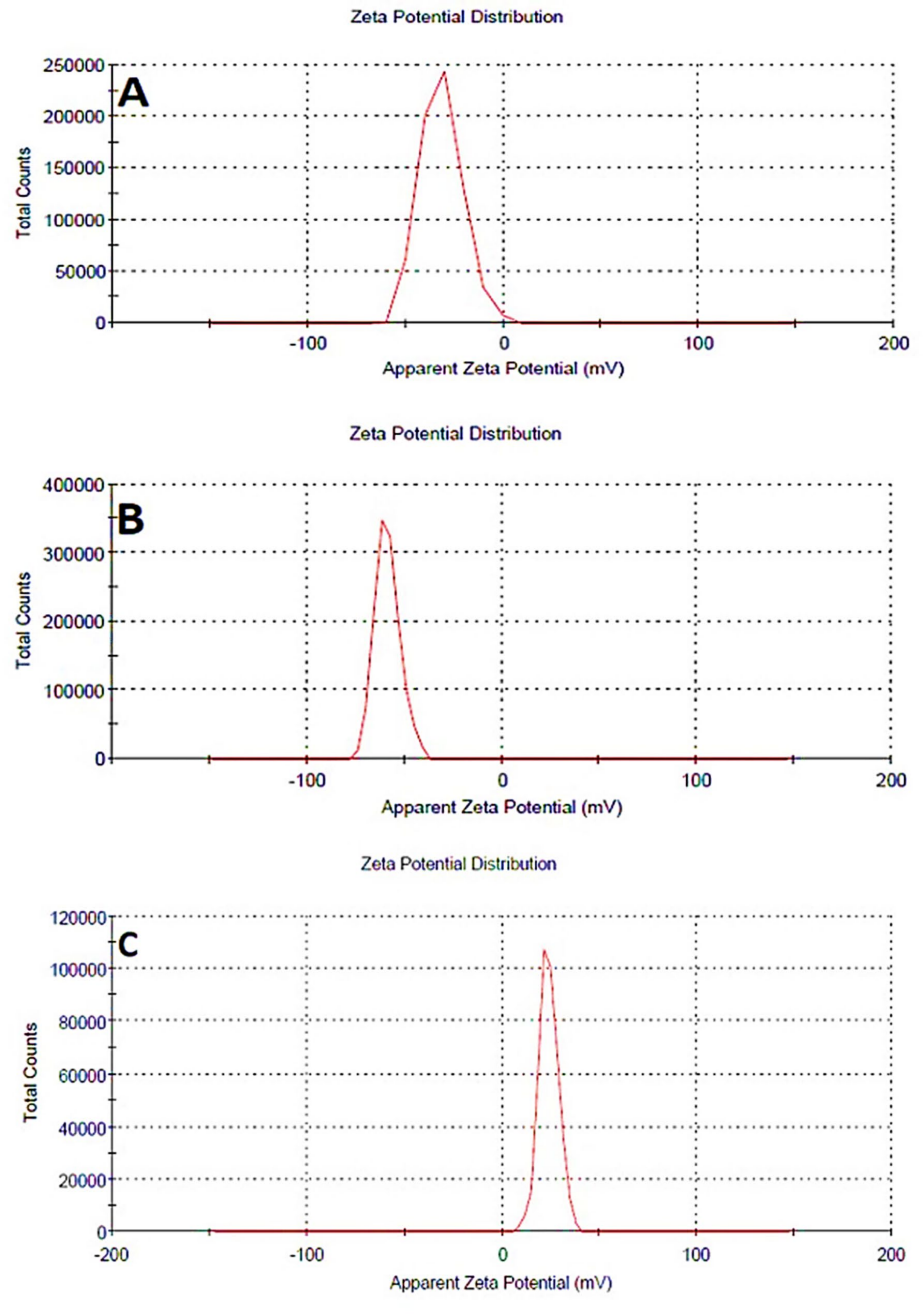
Zeta potential for (**A**) empty soy lecithin liposomal sample, (**B**) β-carotene-encapsulated liposomes, and (**C**) positively charged liposomal β-carotene

According to other findings, negative zeta potential (−31.9 ± 10.7) have shown in blank liposomes [27–29]. Liposomal β-carotene had higher negative zeta potential (−58.8 ± 6.5 mV) than empty liposomes due to the incorporation of β-carotene into the liposomal membranes (Fig. 5). Nanoparticles having zeta potential values larger than positive 30 mV or less than negative 30 mV are more stable in general. The presence of SA in liposomal membranes appears to decrease the density of negative charge, resulting in a positive zeta potential (+ 23.9 ± 5.11 mV).

The results of particle size and zeta potential measurements of various liposomal formulations are summarized in Table 1.

Due to altered interactions between the encapsulated drugs and liposomes, the characterization of DSC was used to study changes in the lipid bilayer phase transition [30, 31].

**Fig. 6:**
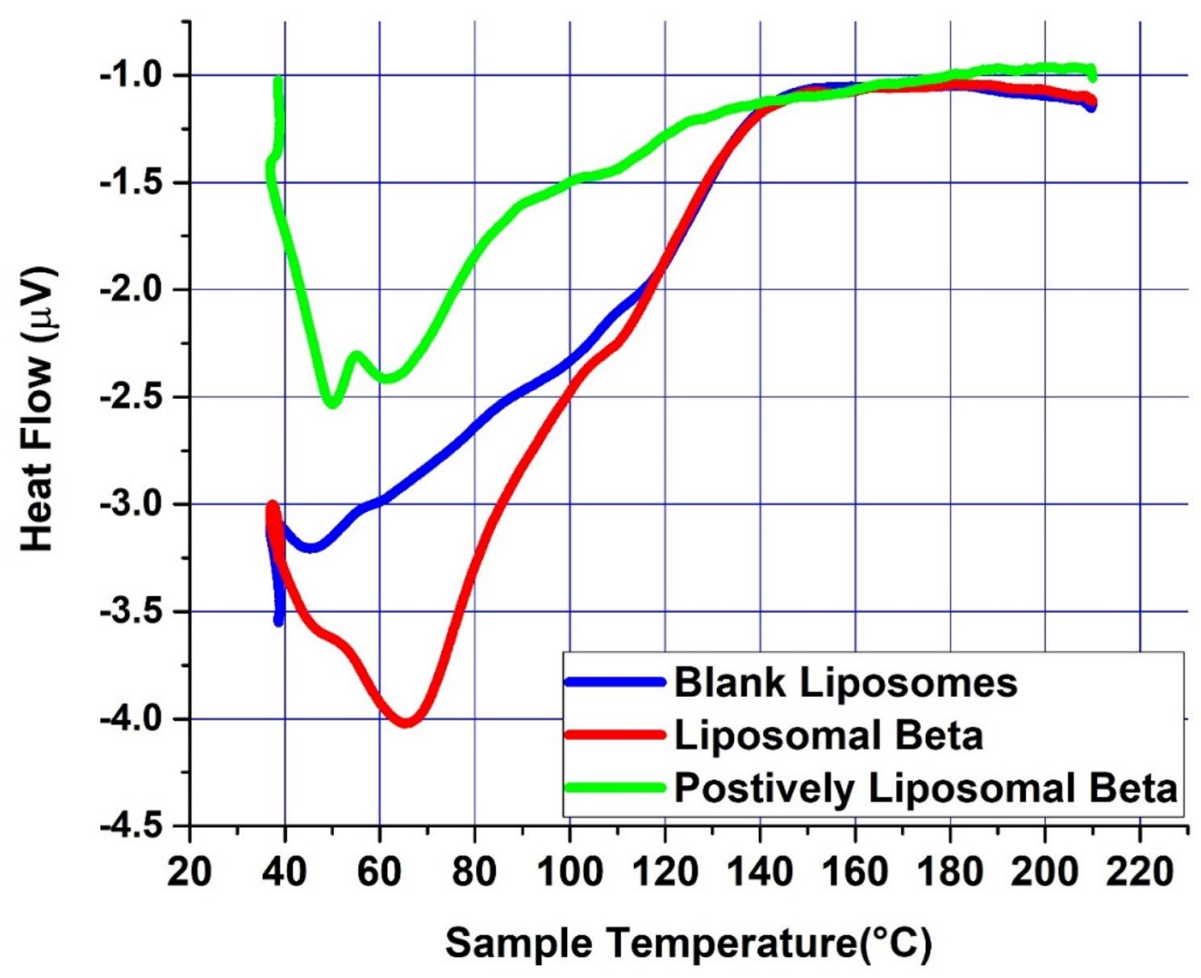
DSC diagrams of liposomes made of pure lecithin, liposomes doped with either β-carotene or β-carotene coated with SA. Liposomal Beta, β-carotene encapsulated in liposomes; Positive Liposomal Beta, positively charged β-carotene liposomes

The lecithin vesicles were used as model membranes because this phospholipid can mimic many characteristics of biological membranes. Pure lecithin vesicles displayed a significant major endothermic peak (T_m_) at 46.34 °C (Fig. 6) the samples were submitted for DSC examination in accordance with the methods described by Spink [32] and Shafaa et al. [33].

The presence of a compound in the membranes of the lecithin may affect the vesicle transition’s thermotropic parameters. The introduction of β-carotene into lecithin liposomes showed a shift to a higher temperature at 67.8 °C compared to the main endothermic peak (T_m_) of empty lecithin where presents at 46.34 °C, which indicates that β-carotene had a substantial effect on the lecithin bilayer of acyl chains creating a conformational order within the phospholipids and increase the transition cooperatively of lipid acyl chains [34, 35]. The increased temperature of the main endothermic peak (T_m_) of empty lecithin suggested that β-carotene incorporation promotes the development of ordered and tight acyl chains.

When SA was incorporated into liposomes previously doped with β-carotene, it had a much greater effect. The SA incorporated into the lipid bilayers most likely conjugated with them, interacted with them extensively, and disrupted them, resulting in the broadening and shifting of the main characteristic endothermic peak of pure lecithin, which occurs at 46.34 °C to a higher temperature (49.8 °C and 64.98 °C). There was the appearance of an additional transition peak at nearly 64.98 °C for the Soy lecithin/β-carotene sample in the presence of SA. It may be assumed that the micro-heterogeneous distribution of β-carotene in the presence of **SA** interacting with liposomes led to the formation of lateral domains.

The insertion of the drug between the polar heads of the lecithin will facilitate the creation of a less orderly liquid crystalline phase than the gel phase and as observed by the DSC, slightly reduce the transition temperature of the gel-to-liquid crystal phase [36, 37]. Using DSC, it has been found that mixtures of lecithin and β-carotene or SA display a single peak, indicating that they are miscible [38].

FTIR has been used to detect any changes in the liposomal membrane structure by examining the wavenumber of different vibrational modes, which has been used to validate some of the modifications found in the current DSC work.

Compared with β-carotene or β-carotene mixed with SA/soy lecithin liposomal samples in the 4000–400 cm^– 1^ region, FTIR spectra of empty lyophilized lecithin liposomes are presented (Fig. 6).

**Fig. 7:**
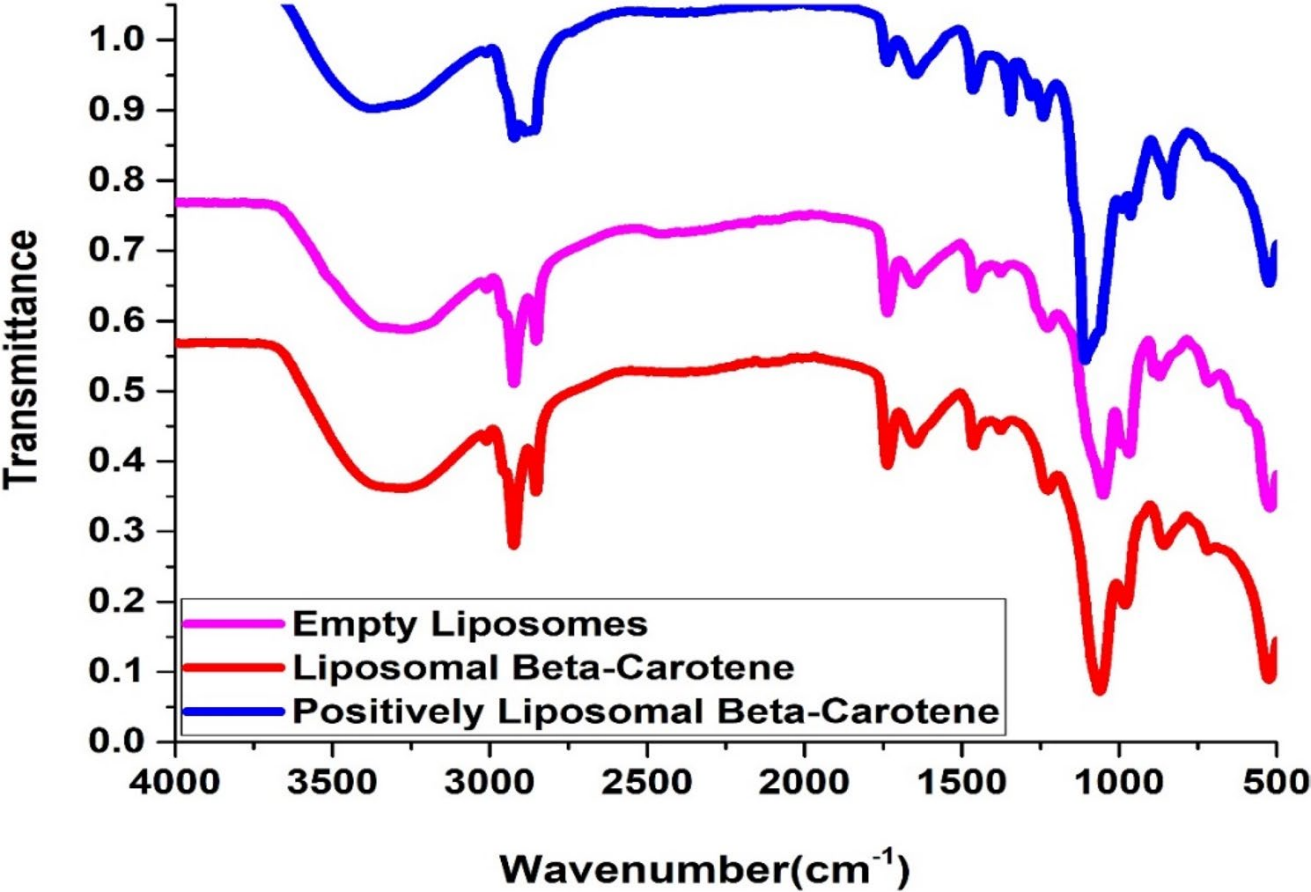
The full FTIR spectra of empty Lecithin, liposomes doped with either β-carotene or β-carotene coated with SA

The main absorption FTIR characteristic peaks was shown by the liposome vesicle spectrum given in [39]. Encapsulation of β-carotene or β-carotene coated with SA into the lecithin liposomes caused a change in the wavenumber of the symmetric CH_2_ stretching bands in the acyl chain (Fig. 7), indicating that β-carotene or β-carotene coated with SA produces a conformational order within the phospholipid’s acyl chains. In other words, they affected the order of the membrane. The peak at 2853.23 cm^− 1^ for the pure lecithin is shifted towards the lower wave number 2849.11 cm^− 1^ or 2847.05 cm^− 1^ for β-carotene liposomes or positively charged liposomal β-carotene, respectively. This may indicate that the number of gauche conformers is decreasing, implying a rise in the bilayer order [40].

The CH_2_ antisymmetric stretching band wavenumber shifted towards the lower wavenumber with the addition of β-carotene or β-carotene coated with SA into liposomes (2917.09 cm^− 1^ or 2915.03 cm^− 1^, respectively) in comparison to those of pure lecithin (2923.27 cm^− 1^) (Fig. 7).

The peaks of CH_2_ symmetric and antisymmetric stretching vibrations have been used as a sensitive alkyl chain ordering indicator. There are substantial changes in the CH_2_ stretching band wavenumber, showing that lutein decreased the number of gauche conformers, suggesting an increase in bilayer conformational order [41, 42].

For the interaction between β-carotene or β-carotene coated with SA and the glycerol backbone near the head group of phospholipids in the interfacial zone, the C = O stretching band is analyzed [43]. As seen from (Fig. 7), the wavenumber value of C = O group at 1736.66 cm^− 1^ remained stable or increased for the liposomal samples containing β-carotene (1736.66 cm^− 1^) or β-carotene coated with SA (1738.72 cm^− 1^), respectively with no evidence of hydrogen bonding formation. In the glycerol backbone region of the soy lecithin molecule, shifts in the contours of ester C = O stretching regulated the degree of hydrogen bond formation. Therefore, any change in the spectrum of this area may be due to the interaction between β-carotene or SA and the apolar/polar interfacial region of the membrane [44].

The interaction of β-carotene or β-carotene coated with SA with the head group of soy lecithin liposomes was investigated using the PO_2_^–^ antisymmetric stretching band at 1231.94 cm^– 1^. As depicted from Fig. 7, the wavenumber was shifted to lower values after the addition of β-carotene (1229.87 cm^– 1^) into lecithin liposomes. This implied the presence of hydrogen bonding between the liposome head group and β-carotene. The decrease in the value of the wavenumber implies that existing hydrogen bonds are strengthened or new hydrogen bonds between the components are formed [40, 44].

The incorporation of β-carotene or β-carotene coated with SA into lecithin liposomal preparation affects the CH_2_ scissoring vibration mode, which is located at 1464.72 cm^– 1^. The wavenumber was moved towards lower values at 1462.66 cm^– 1^ after encapsulation of β-carotene into lecithin liposomes. This suggests that β-carotene molecules do not serve as small spacers for the polar head group Table 2.

**Table 2:** The chemical shifts observed for β-carotene or β-carotene coated with SA after the incorporation into soy lecithin liposomes

Peak assignment	Wavenumber (cm^–1^)	Wavenumber (cm^–1^)		
		Empty Liposomes	Liposomal β-carotene	positively charged liposomal β-carotene
Symmetric stretching vibration of CH_2_ in acyl chain	(2800–2855)	2853.23	2849.11	2847.05
Antisymmetric stretching vibration of CH_2_ in acyl chain	(2900–2925)	2923.27	2917.09	2915.03
Carbonyl stretching vibration C=O	(1730–1740)	1736.66	1736.66	1738.72
CH_2_ scissoring vibration	(1456–1470)	1464.72	1462.66	1464.72
Antisymmetric PO_2_^–^stretching vibrations	(1200–1300)	1231.94	1229.87	1242.23

**Fig. 8:**
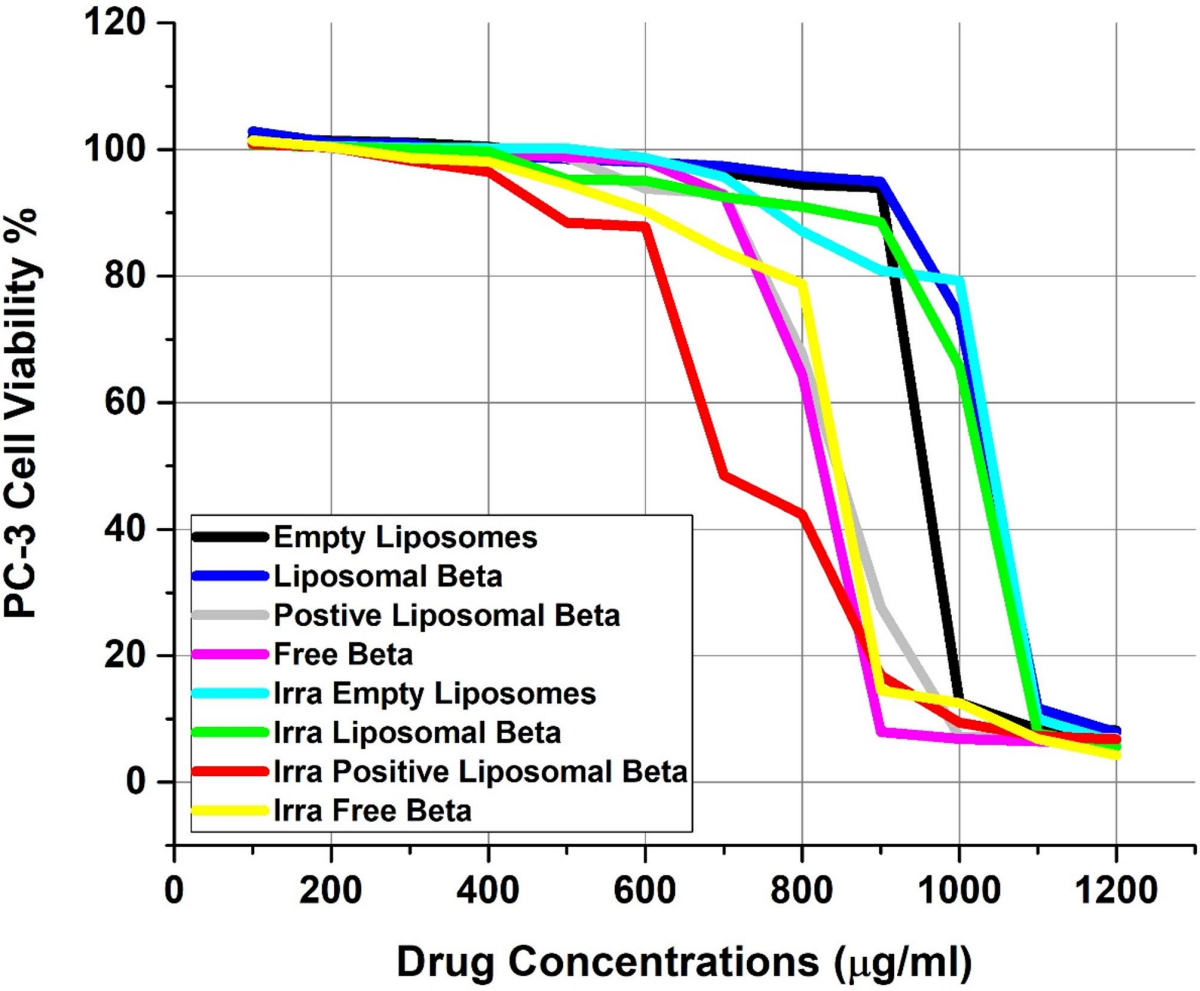
In vitro cytotoxicity of free β-carotene, empty liposomes, liposomal β-carotene, and positively charged liposomal β-carotene in the absence or presence of external gamma-irradiation (at dose 5 Gy) against prostate carcinoma (PC-3) cell line; incubated for 48 h with different drug concentrations starting from 100 to 1200 µg/ml. The MTT assay was used to measure cell viability. The results are the average standard error of three replicate studies. Abbreviation: Liposomal Beta, β-carotene encapsulated in liposomes; Positive Liposomal Beta, positively charged β-carotene liposomes; Free Beta, free (unencapsulated) β-carotene; Empty Liposomes, liposomes without β-carotene; Irra Empty Liposomes, irradiated empty liposomes; Irra Liposomal Beta, irradiated β-carotene liposomes; Irra Positive Liposomal Beta, irradiated positively charged β-carotene liposomes; Irra Free Beta, irradiated free β-carotene

Using the Prostate carcinoma (PC-3) cell line, the drug delivery system’s effectiveness was evaluated in the presence or absence of external gamma-irradiation using the cell viability MTT assay (In-Vitro cytotoxicity) at various drug concentrations of free β-carotene, empty liposomes, liposomal β-carotene, and positively charged liposomal β-carotene [21]. Untreated cells served as monitors at each drug’s zero con centration. Separately, for 48 h, the PC-3 cancer cell line was cultured at various drug concentrations using the same protocol, ranging from 100 to 1200 µg/ml (Fig. 8). After 48 h, the experiment was stopped, and cell viability was assessed.

Free β-carotene followed by positively charged liposomal β-carotene showed the highest rate of cytotoxicity against the PC-3 cell line when treated with the same sequence of different concentrations available for other drugs, even in the absence of external gamma-irradiation. 48 h after treatment, PC-3 treated cells showed cell viability at the maximal free β-carotene concentration (1200 µg/ml) of roughly 6.37%. At the same concentration (1200 µg/ml), the cell viability for liposomal β-carotene-treated cells was approximately 7.90%, while for positively charged liposomal β-carotene -treated cells, it was approximately **7**%. The drug’s entrapment within several lipoidal domains of vesicles is the cause of its diminished efficacy in the lipo-solubilized state.

Interestingly, empty liposomes exhibited considerable reduction in the cell viability against PC-3 cell line when treated with the same concentration (1200 µg/ml). The vitality of the cells was at 8.15%. This indicates that the cells were harmed by the high concentration of lecithin liposomes. Rashidinejad et al. (2014) [8] reported that angiogenesis and cellular proliferation in triple-negative breast cancer (TNBC) associated tumors were inhibited by DPPA liposomes. The current study presents initial evidence that could validate the therapeutic potency of DPPA for the treatment of TNBC.

Cytotoxic activity among various drug formulations at higher concentrations (1200 µg/ml) displayed the order of Free β-carotene > positively charged liposomal β-carotene > liposomal β-carotene > empty liposomes according to Table 3; Fig. 8. Cell viability is biologically capped at 0–100% (0% = cell death, 100% = survival similar to control); experimental results over 100% are occasionally observed, typically a reflection of greater proliferation or metabolic stimulus (hormesis), normalization to the control, or technical variation in MTT assays.

**Table 3:** Cell viability (%) prostate carcinoma (PC-3) cell line treated with different β-carotene formulations (free, liposomal, and positively charged liposomal), with and without irradiation, at varying concentrations (µg/mL)

Concentration	Empty Liposomes	Liposomal Beta.	Positive Liposomal Beta.	Free Beta.	Irra. Empty Liposomes	Irra. Liposomal Beta.	Irra. Positive Liposomal Beta.	Irra. Free Beta.
1200	8.2	7.9	7.0	6.4	6.3	5.6	6.8	4.3
1100	8.4	11.7	7.0	6.5	10.0	7.8	7.3	6.9
1000	12.6	73.7	7.2	6.9	79.2	65.7	9.4	12.5
900	94.0	94.9	27.7	8.0	80.9	88.5	16.9	14.5
800	94.5	95.8	67.6	64.4	87.0	90.9	42.3	78.8
700	96.4	97.3	93.0	92.8	95.7	92.5	48.5	83.8
600	98.0	98.0	93.8	98.2	98.7	95.1	87.8	90.3
500	98.9	98.5	98.8	98.8	100.1	95.3	88.5	94.5
400	100.5	99.2	100.1	99.5	100.2	99.7	96.5	98.1
300	101.1	100.9	100.1	99.7	100.3	100.1	98.3	98.7
200	101.4	101.1	100.3	100.3	100.5	100.4	100.4	100.4
100	101.9	102.9	101.5	101.4	100.9	100.7	100.9	101.4

Notably, under certain non-irradiated conditions, free β-carotene demonstrated greater cytotoxicity than its liposomal counterpart. This differential response may be attributed to factors such as suboptimal entrapment efficiency or the inherently slower release kinetics associated with liposomal encapsulation, which can delay the availability of bioactive β-carotene to cells. While the liposomal formulation confers several advantages overall, including improved stability and controlled delivery, this observation represents a potential limitation of the present study and warrants further investigation.

At the lower concentration at 800 µg/ml, PC-3-treated cells with free β-carotene showed cell viability 64.43% compared to its liposomal form or positively charged liposomal β-carotene of about 95.85% or 67.64%, respectively, Fig. 8.

**Fig. 9:**
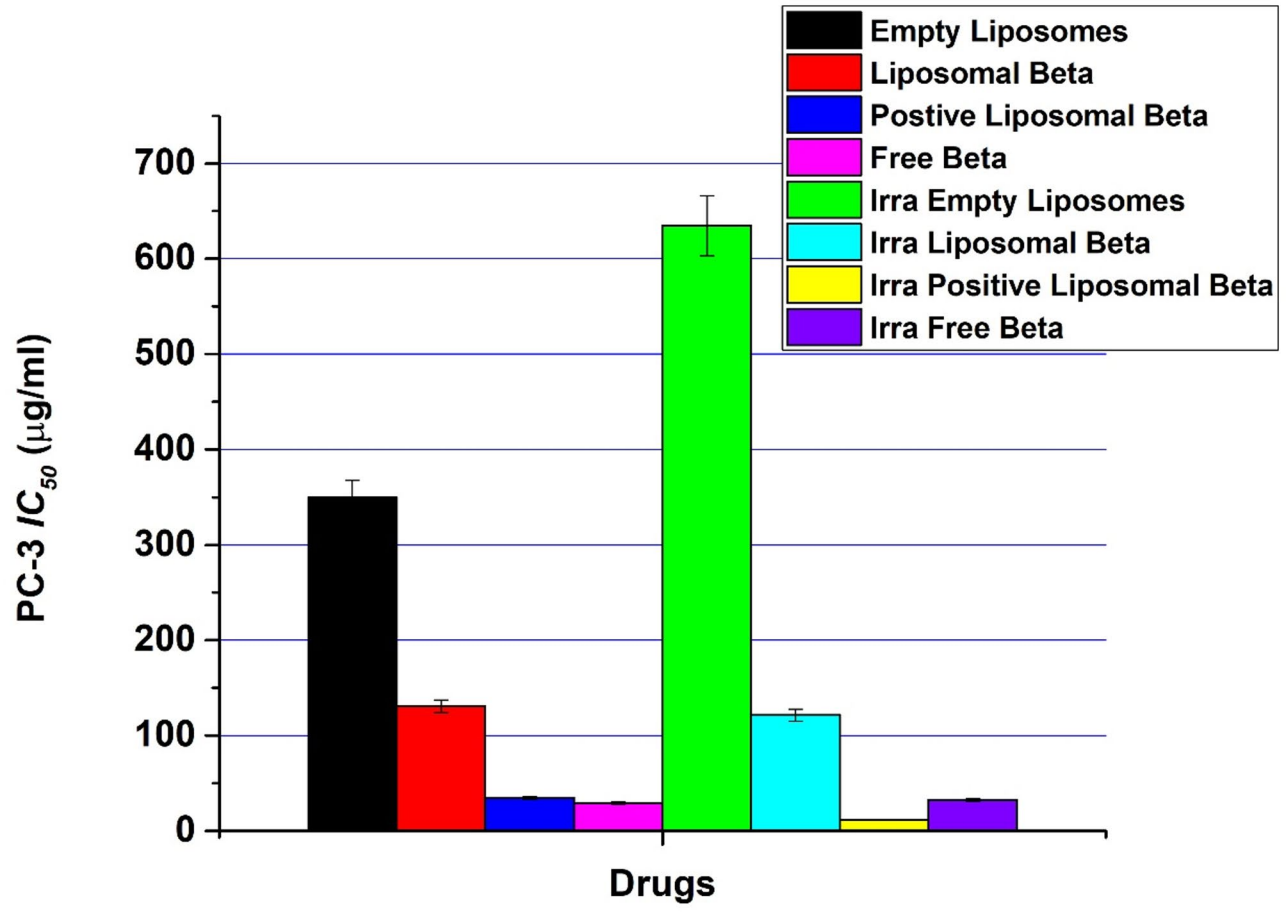
IC_50_ values for free β-carotene, empty liposomes, liposomal β-carotene, and positively charged liposomal β-carotene in the absence or presence of external gamma-irradiation (at dose 5 Gy) against prostate carcinoma (PC-3) cell line by using MTT assay, 48 h post-treatment

In the absence of external gamma-irradiation, the IC_50_ value for free β-carotene in cytotoxic assay with PC-3 treated cells was 29.24 µg/ml, while liposomal β-carotene for PC-3 treated cells was counted as 130.69 µg/ml. IC_50_ was 34.74 µg/ml for PC-3 treated cells with positively charged liposomal β-carotene. Based on the above results and depending on the cancer cells type, free β-carotene showed the highest therapeutic efficacy against PC-3 cell line Fig. 9. It can be noticed that the IC_50_ of empty liposomes was 350.06 µg/ml in cytotoxic assay with PC-3.

The proliferation/viability of PC-3 cells was assessed by MTT assay after the incubation with the tested chemotherapeutic agents in the presence of external irradiation of cells with radiation dose of 5 Gy using a ^137^Cs, respectively (Fig. 8). The cytotoxic effect of the tested chemotherapeutic agents increased with radiation dose of 5 Gy. The toxicity of all tested chemotherapeutic agents increased as the drug concentration increased in a concentration-dependent manner. At the maximum concentration (1200 µg/ml) combined with irradiation of cells at 5 grays (Fig. 8), the toxic effect of free β-carotene followed by liposomal β-carotene markedly decreased the cell viability to 4.31% and to 5.61%, respectively. In this cytotoxicity test, the positively charged liposomal β-carotene caused less death of viable cells than liposomal β-carotene or free β-carotene, where the cell viability was 6.77%. Empty liposomes succeeded in producing more toxicity to viable cells in the presence of 5 Gy irradiation dose and exhibited enhanced viability from 8.15%. to about 6.34%.

At the lower concentration at 800 µg/ml that combined with irradiation of cells at 5 grays, PC-3 treated cells with free β-carotene displayed cell viability 78.77% relative to their liposomal form of about 90.93%, while 42.31% of the cell remained viable for positively charged liposomal β-carotene (Fig. 8).

Sensitization rates were measured at various treatment modalities to investigate the relationship between the concentration of the tested different formulations of β-carotene or β-carotene coated with SA, the radiation dose, and the β-carotene or β-carotene coated with SA sensitization effect.

It is obvious that the combination therapy regimen (free β-carotene or empty liposomes or liposomal β-carotene or positively charged liposomal β-carotene + radiation) has a more effective anti-cancer effect than the treatment regimen without radiation. This suggests that a combination of chemo and radiation therapy is more successful than a single therapy regimen.

Cytotoxic activity among various drug formulations at the maximum concentration (1200 µg/ml) that combined with irradiation of cells at 5 grays displayed the order of Free β-carotene > liposomal β-carotene > empty liposomes > positively charged liposomal β-carotene according to Fig. 8.

The IC_50_ value for free β-carotene in cytotoxic assay with PC-3 treated cells in the presence of gamma-irradiation at doses of 5 grays was 32.46 µg/ml, while liposomal β-carotene for PC-3 treated cells was counted as 121.48 µg/ml. IC_50_ was 11.66 µg/ml for PC-3 treated cells with positively charged liposomal β-carotene. Based on the above results and depending on the cancer cells type, positively charged liposomal β-carotene showed the highest therapeutic efficacy against PC-3 cell line Fig. 9. It can be noticed that the IC_50_ of empty liposomes was 634.73 µg/ml in cytotoxic assay with PC-3.

**Fig. 10:**
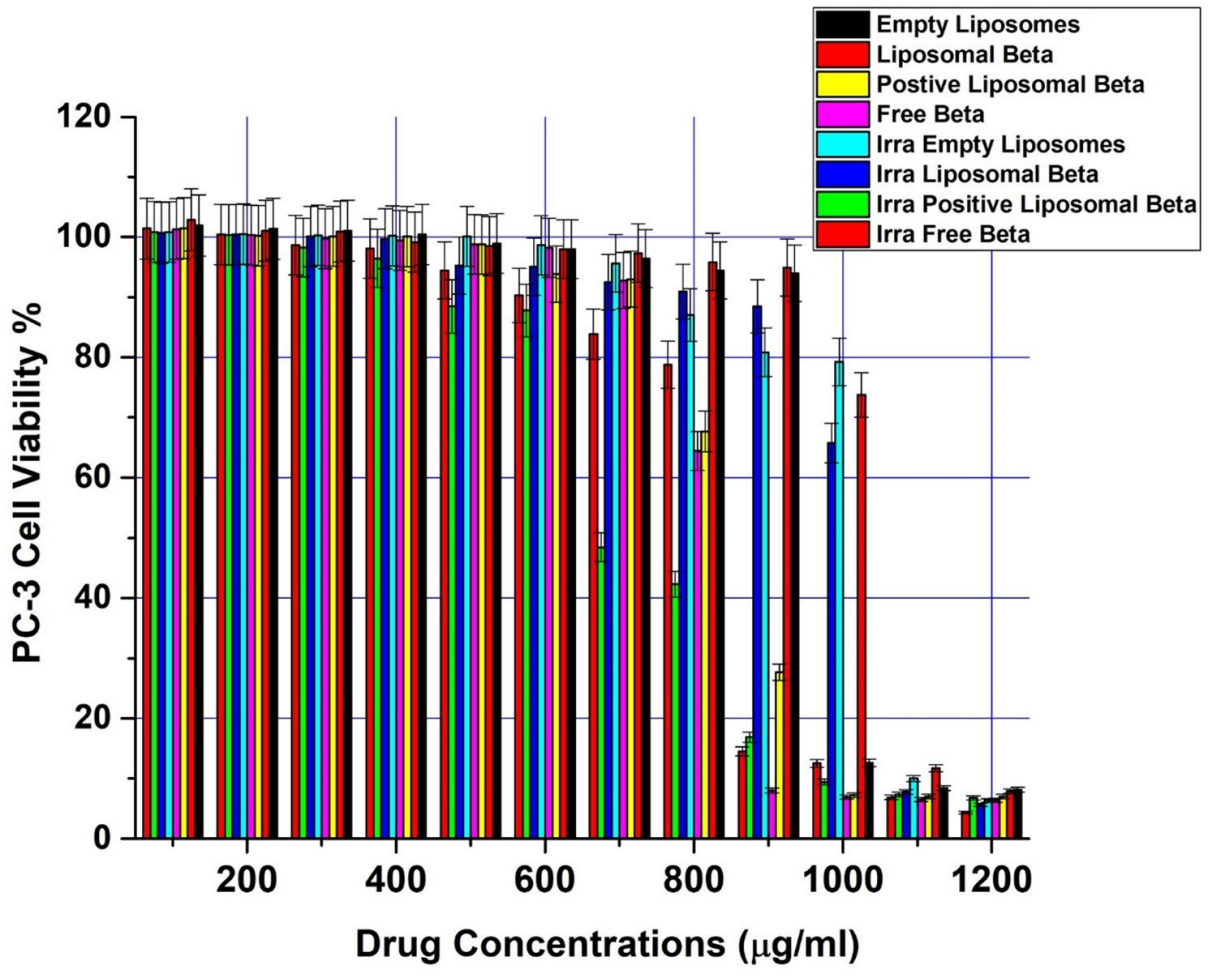
Summarized cytotoxicity values obtained for free β-carotene, empty liposomes, liposomal β-carotene, and positively charged liposomal β-carotene in the absence or presence of external gamma-irradiation (at dose 5 Gy) against prostate carcinoma (PC-3) cell line; incubated for 48 h with different drug concentrations starting from 100 to 1200 µg/ml. The MTT assay was used to measure cell viability. The results are the average standard error of three replicate studies.

Figure 10 represents the summarized data of cytotoxicity values obtained for free β-carotene, empty liposomes, liposomal β-carotene, and positively charged liposomal β-carotene in the absence or presence of external gamma-irradiation (at dose 5 Gy) against prostate carcinoma (PC-3) cell line by using MTT assay

Positively charged liposomal β-carotene can act as a radio-sensitizer, increasing the amount of radiation delivered to the tumor site. Radio-sensitizers are used in radiation therapy to increase the radiation absorbed dose to the tumor site by increasing the intrinsic radio-sensitivity of cancer cells. Treatment may be carried out with a lower dose of radiation, reducing the adverse effects on normal cells [10].

The positively liposomal form of β-carotene exhibits a significantly different cell-killing effect than free form. We suggested that the likelihood of nanoparticle aggregation may be enhanced by adding β-carotene into soy lecithin liposomes. This is predominant when there is a large concentration of nanoparticles, which causes the diameters of the particles to rise (see Table 1). According to research by Xiao-Dong and colleagues, radiosenstization is significantly influenced by the size of the nanoparticles [45]. Consequently, especially at higher concentrations, the radiosenestration impact of the drug-loaded nanoparticle was somewhat less than that of the empty one. The chemotherapeutic impact will therefore make up for this decline.

The role of chemotherapy in conjunction with radiation therapy is critical because it inhibits cell repair and regulates metastasis, in addition to its efficacy with localized radiation therapy. Furthermore, the fact that β-carotene is trapped in several lipoidal domains of the nanoparticles may account for their enhanced ability to kill in the lipo-solubilized state.

**Fig. 11:**
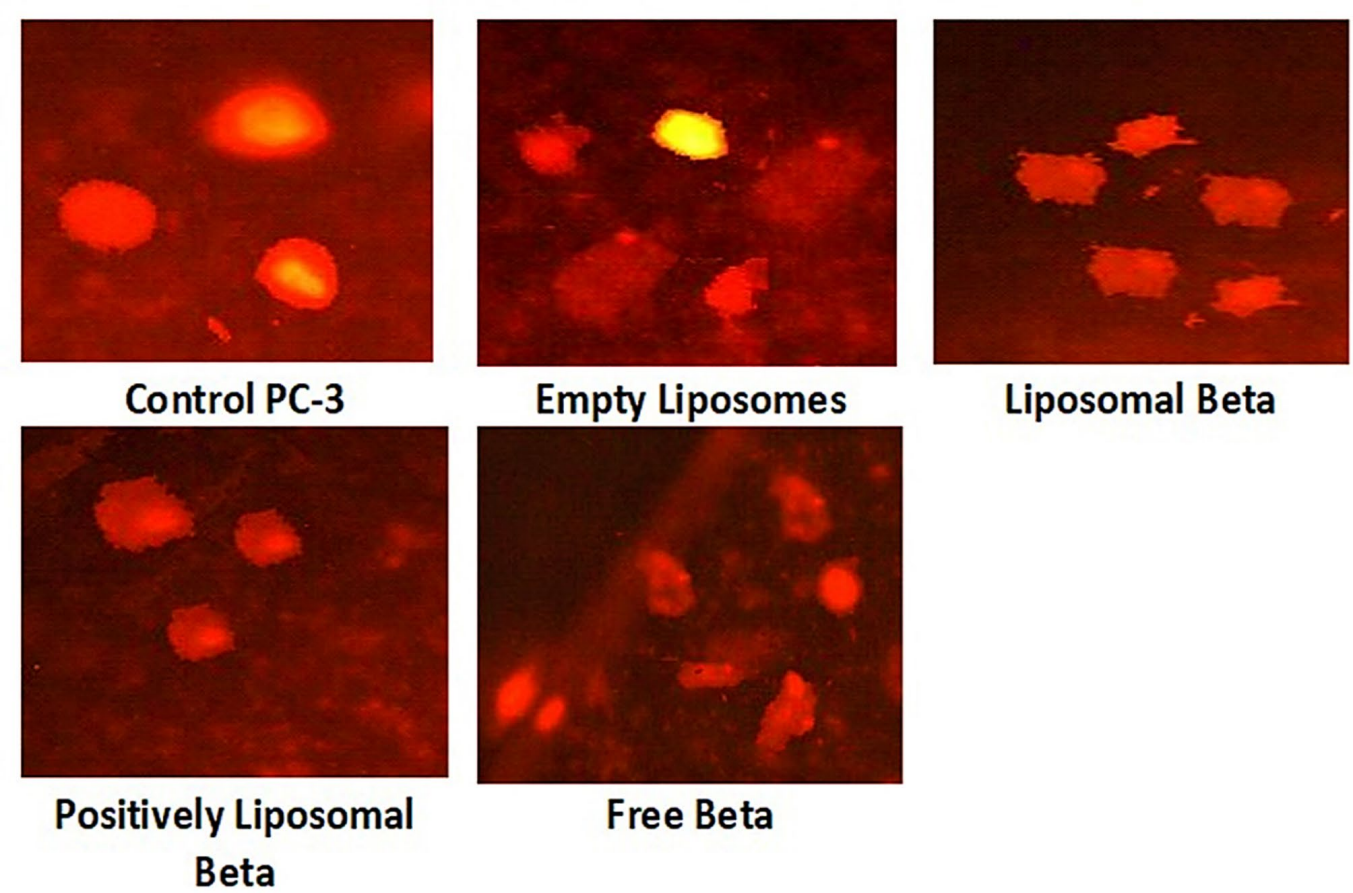
Comet assay images of PC-3 cell line (evaluation of DNA damage induced by free β-carotene, empty liposomes, liposomal β-carotene, and positively charged liposomal β-carotene compared to control PC-3)

For all the formulations added to the PC-3 cell line, the analysis of the comet assay photographs is given in Fig. 11. The intact DNA without migration was represented by the red round spot in the photograph, while the comet-shaped region adjacent to the nucleus represented breaks of DNA small enough to move in the gel, indicating the undamaged control cell. The DNA was tightly packed, and a typical nucleus was retained in its circular disposition. The photograph of the comet for the profile of nuclear DNA which was altered with the presence of a fluorescence streak extending from the nucleus. Damaged DNA-containing cells looked like a comet with a bright head and tail.

In the absence of external gamma-irradiation, PC-3 cells treated with liposomal β-carotene, Fig. 11 shows a higher intensity of the comet tail than those treated with β-carotene alone. The high intensity of the tail of the comet compared to the head means that there are a significant number of double-strand breaks. An increased percentage of mortality was observed for PC-3 cells treated with liposomal β-carotene. These results support the assumption that a nano-sized delivery system can reach cancer cells more effectively at a faster rate than its large-sized counterparts. As the tail length and density represent the amount of single strand breaks in the DNA, a quantitative measure of the damaged DNA is given by the percentage of DNA in the tail. The increased mean tail moment is also indicative of injury to DNA.

**Fig. 12:**
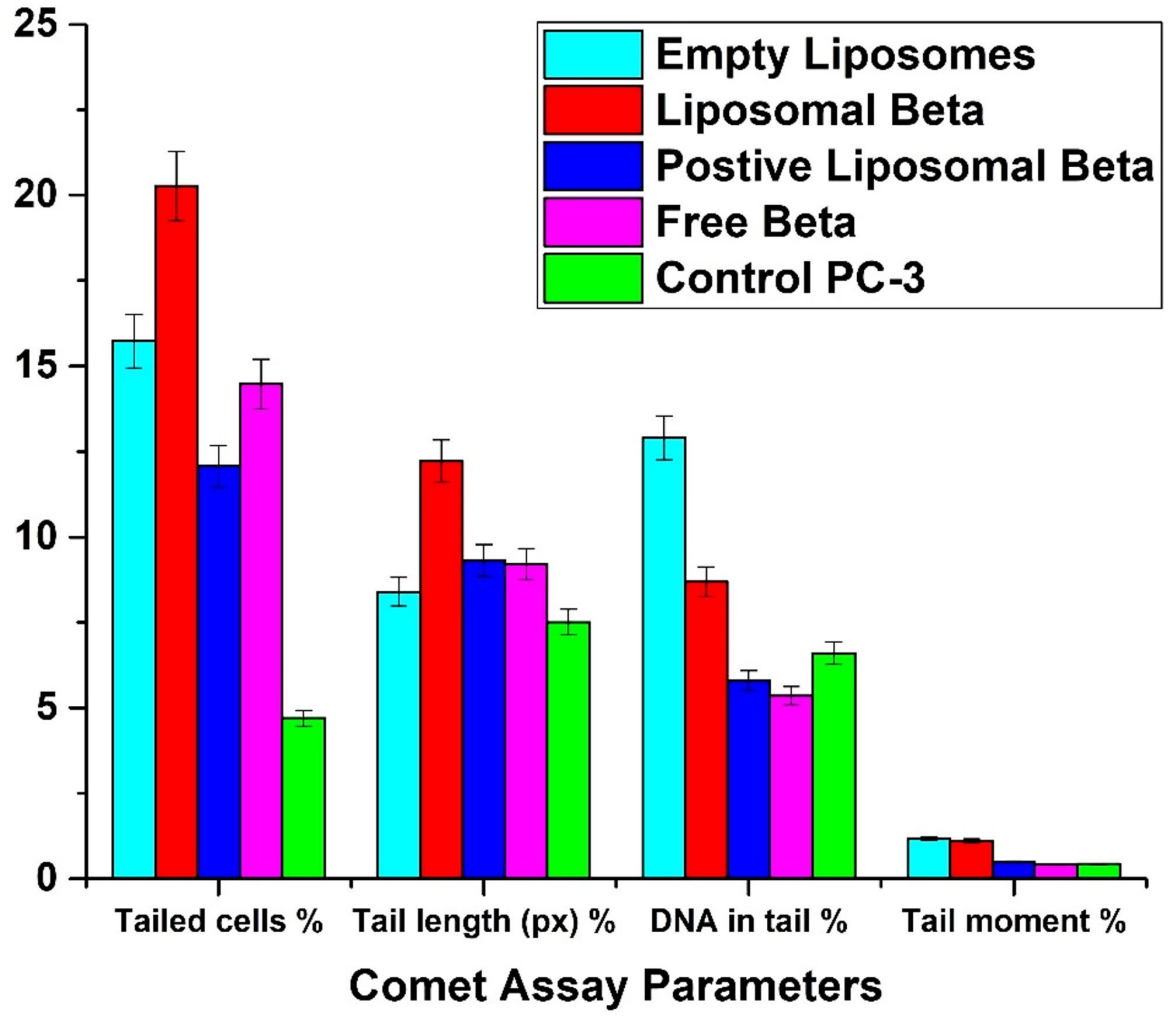
Indicates the comet assay parameters (percentage tailed cells, tail length, percentage tailed DNA, and tail moment) for the PC-3 control cell line and post-treatment groups, and the differences between the control and the post-treatment groups

Figure 12 displays the comet assay parameters (percentage of tail cells, tail length, percentage of tail DNA, and tail moment) for the PC-3 control cell line and post-treatment with free β-carotene, empty liposomes, liposomal β-carotene, and positively charged liposomal β-carotene separately, as opposed to the control group, and the variations between the control and post-treatment groups. The results showed that for the liposomal β-carotene group, all comet assay parameters were significantly increased (*P* < 0.05) relative to the control values.

**Fig. 13:**
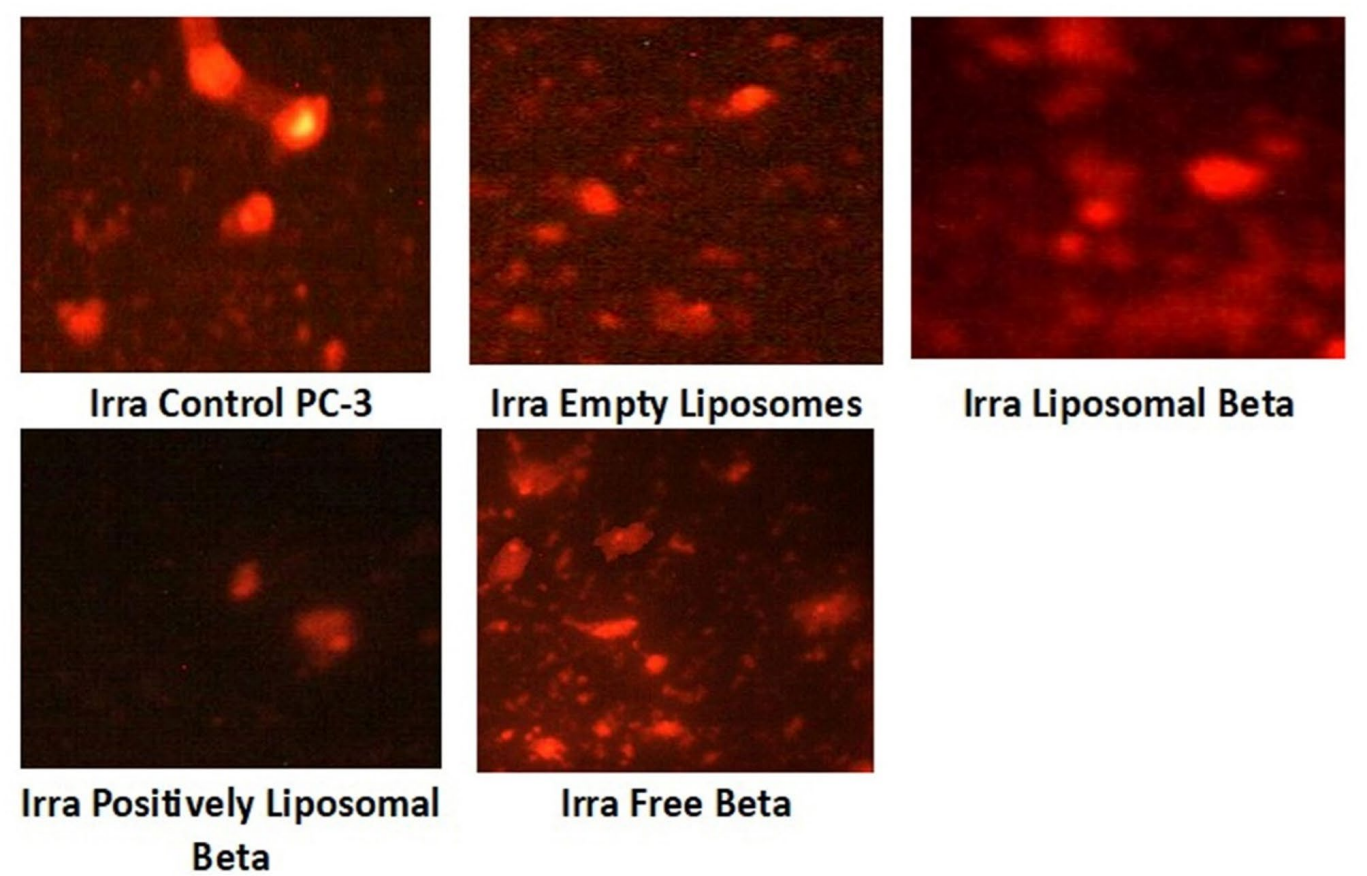
Comet assay images of 5 grays radiation-exposed PC-3 cell line (evaluation of DNA damage induced by free β-carotene, empty liposomes, liposomal β-carotene, and positively charged liposomal β-carotene compared to control PC-3)

Figure 13 demonstrates that the comet tail is more intense in radiation-exposed PC-3 cells treated with positively charged liposomal β-carotene than in cells treated with β-carotene alone. A considerable number of double-strand breaks are present because the comet’s tail is much more intense than its head. PC-3 cells treated with positively charged liposomal β-carotene showed a higher rate of mortality. These findings provide credence to the theory that a delivery system at the charged nanoscale can more efficiently and quickly target different types of cells than one at the vast scale. Since the length and density of the tail indicate the number of single-strand breaks in the DNA, the percentage of DNA in the tail provides a quantitative assessment of the damaged DNA. The increased mean tail moment is also indicative of injury to DNA.

**Fig. 14:**
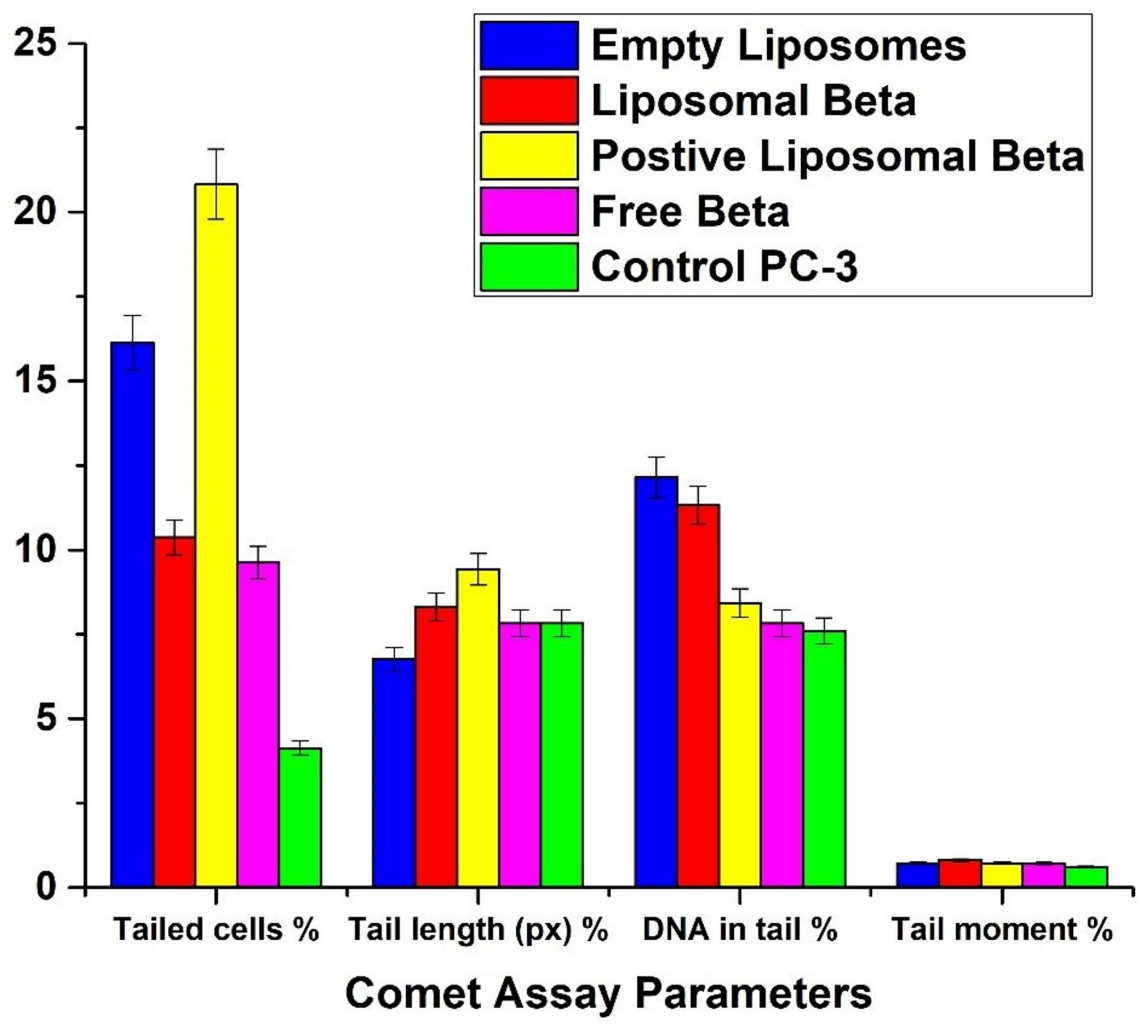
Indicates the comet assay parameters (percentage tailed cells, tail length, percentage tailed DNA, and tail moment) for the PC-3 control cell line and post-treatment groups, and the differences between the control and the post-treatment groups

In contrast to the control group, Fig. 14 shows the deviations between the control and post-treatment groups as well as the comet assay parameters (tail length, tail moment, percentage of tail cells, and percentage of tail DNA) for the radiation-exposed cell line and post-treatment with free β-carotene, empty liposomes, liposomal β-carotene, and positively charged liposomal β-carotene separately. The findings demonstrated that, in comparison to the control values, All Comet assay parameters were significantly higher in the positively charged liposomal β-carotene group, as determined by one-way ANOVA (*P* < 0.05).

**Fig. 15:**
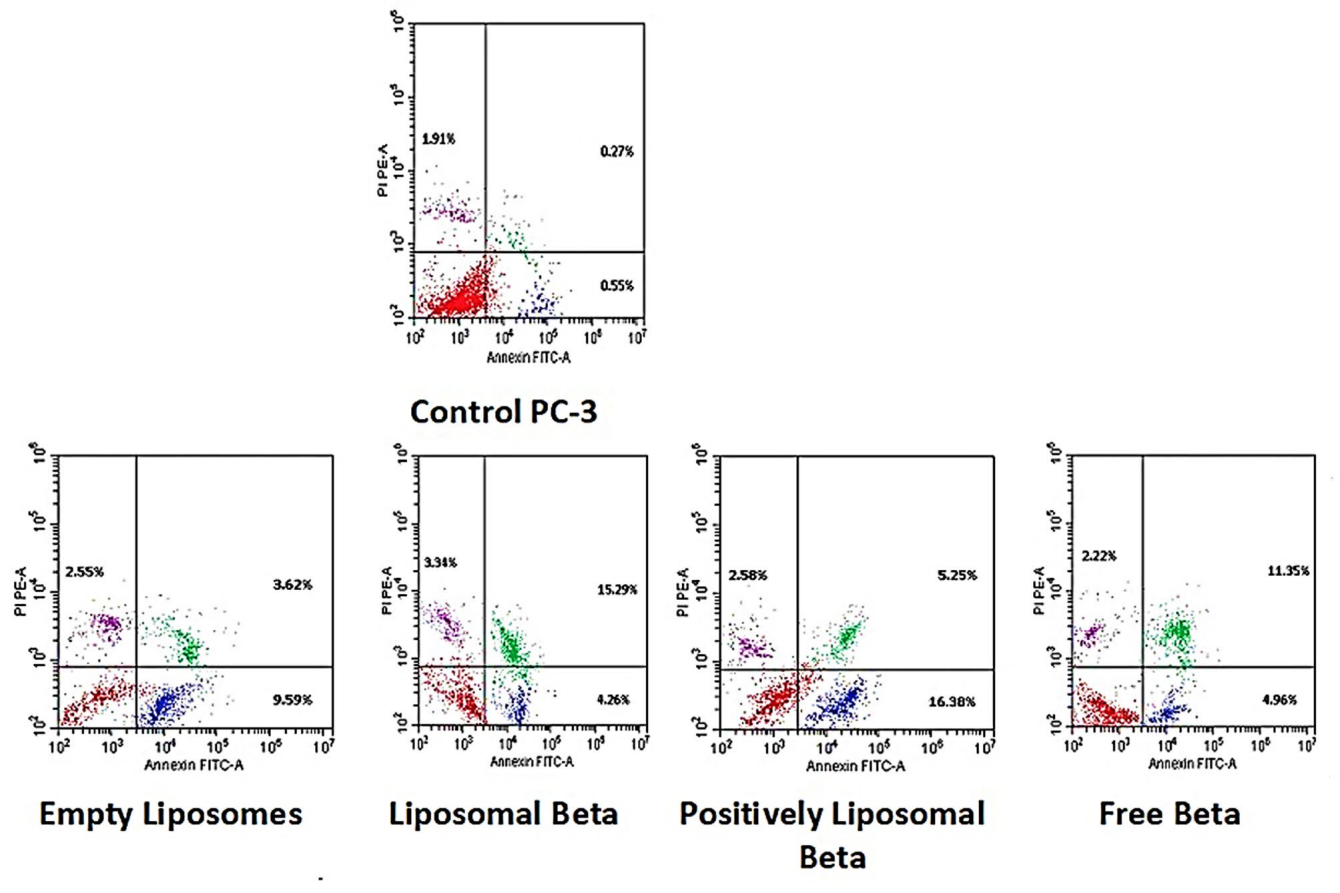
Flow cytometric analysis of PC-3 cells: Control PC-3, treated with the IC_50_ concentration of free β-carotene, empty liposomes, liposomal β-carotene, and positively charged liposomal β-carotene for 48 h

Figures 15 and 17 illustrates the different stages of apoptosis induced by free β-carotene, empty liposomes, liposomal β-carotene, and positively charged liposomal β-carotene separately in PC-3 cells, as analyzed using Annexin V-FITC/PI staining. The graph shows the percentage of cells in each stage of apoptosis, including viable cells, early apoptotic cells, late apoptotic cells, and necrotic cells, for the IC_50_ concentration of all tested samples. The results indicate that the highest percentage of early and late apoptosis (25.21%) was observed when PC3 cells were exposed to the IC_50_ concentration of positively charged liposomal β-carotene followed by liposomal β-carotene (22.89%). Additionally, untreated cells had a very small percentage of the distinctive apoptotic features.

It was observed in our study that a significant increase in apoptosis was induced when the IC_50_ concentration of positively charged liposomal β-carotene was administered to PC-3 cancer cells, compared to free β-carotene only suggesting a correlation between lower positively charged liposomal β-carotene resistance and increased apoptosis. To evaluate the effect of positively charged liposomal β-carotene on cell viability, the expression of the apoptotic marker Annexin V was measured. Flow cytometric analysis revealed that after 48 h of exposure to positively charged liposomal β-carotene, higher levels of total apoptotic cell populations (Q2 + Q4) were exhibited by the PC-3 cells compared to the control cells. This indicates that the effects of positively charged liposomal β-carotene were less resisted by the PC-3 cells.

**Fig. 16:**
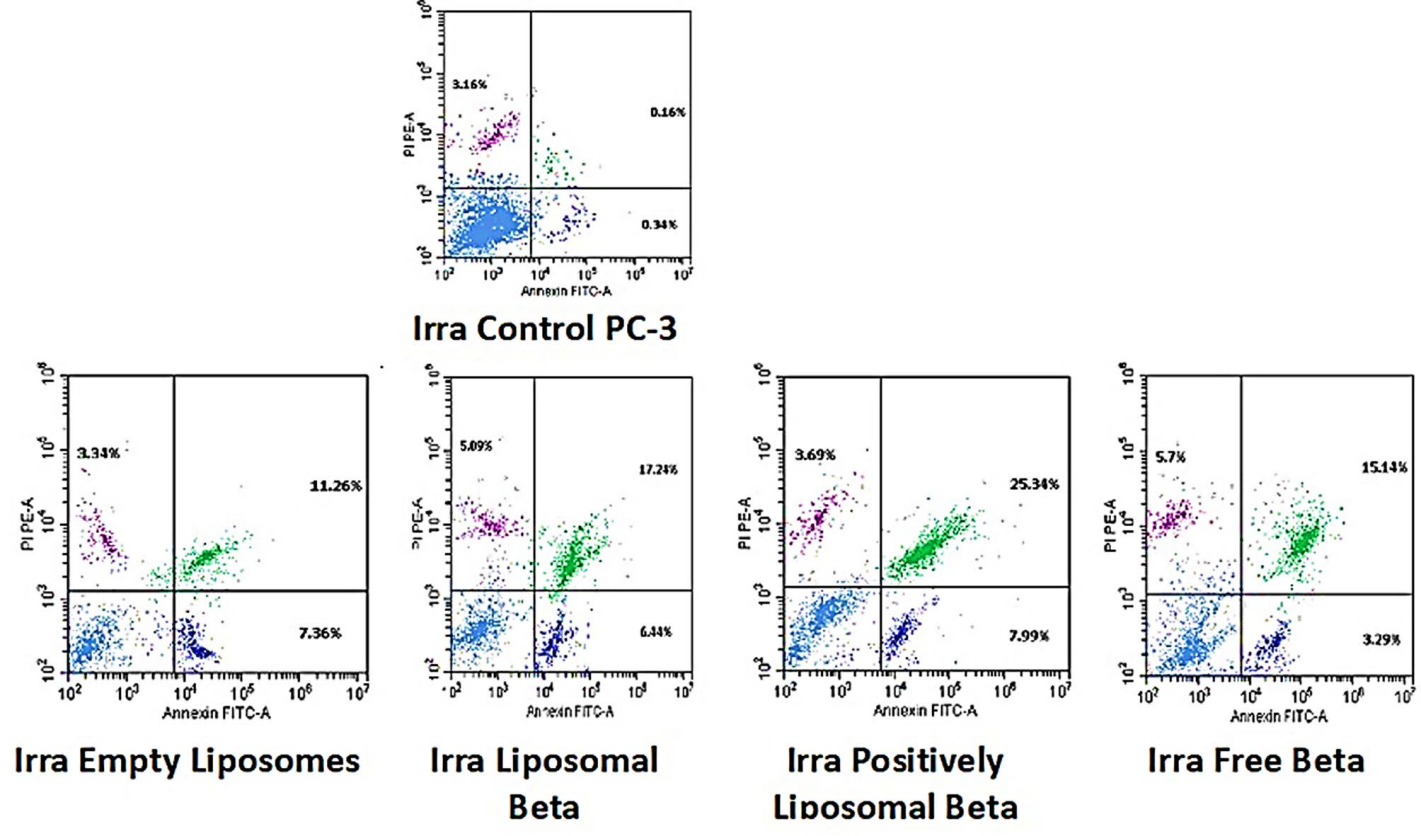
Flow cytometric analysis of radiation-exposed PC-3 cells at 5 grays: Control PC-3, treated with the IC_50_ concentration of free β-carotene, empty liposomes, liposomal β-carotene, and positively charged liposomal β-carotene for 48 h

**Fig. 17:**
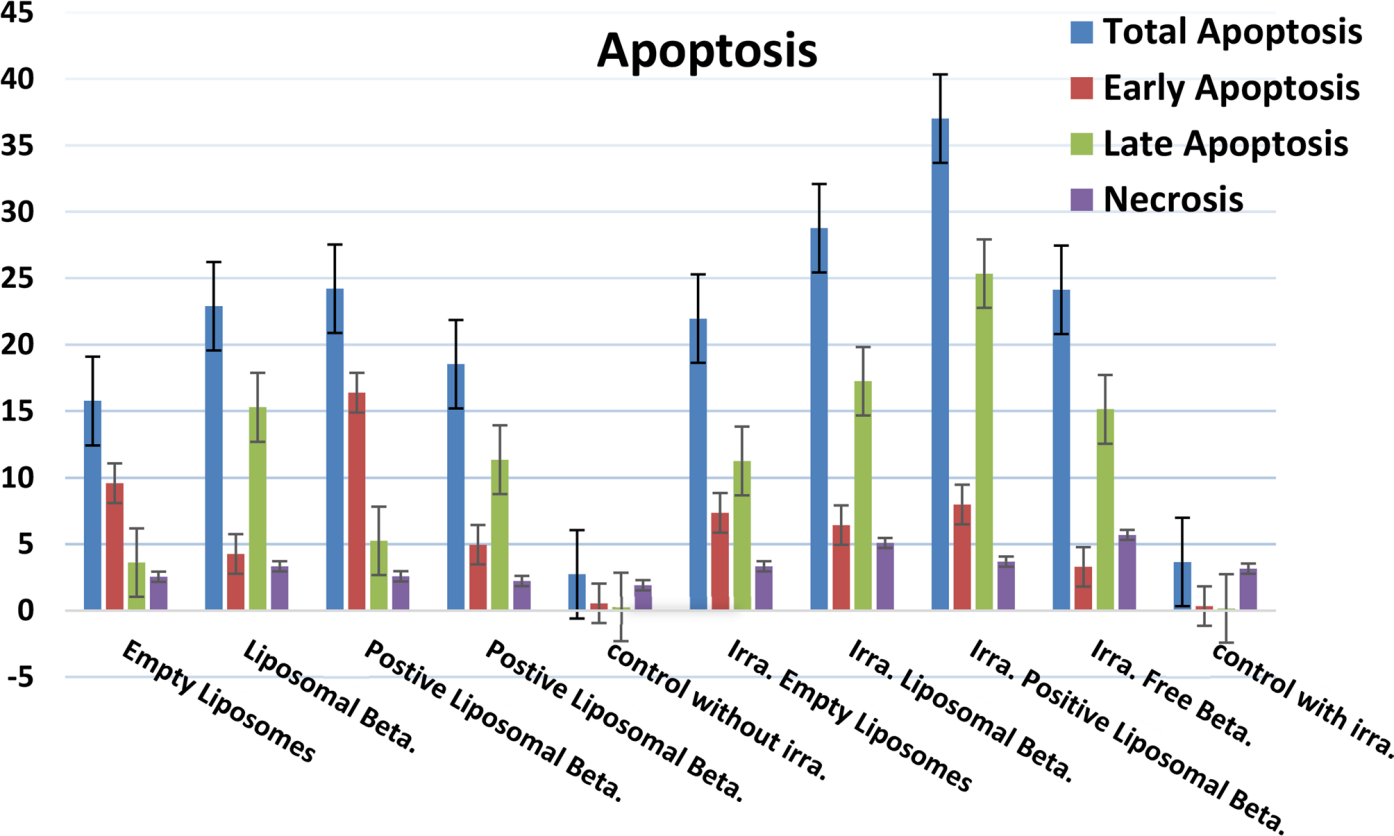
Flow cytometry analysis of apoptosis in PC-3 cells after 48 h incubation with IC₅₀ concentrations of free β-carotene, empty liposomes, liposomal β-carotene, and positively charged liposomal β-carotene, with and without irradiation. Data are expressed as percentages of total apoptosis, early apoptosis, late apoptosis, and necrosis (mean ± SD)

In PC-3 cells irradiated with 5 grays, Figs. 16 and 17 shows the various stages of apoptosis brought on by free β-carotene, empty liposomes, liposomal β-carotene, and positively charged liposomal β-carotene independently. PC-3 cells subjected to the IC_50_ dose of positively charged liposomal β-carotene, followed by liposomal β-carotene (28.77%), showed the highest proportion of early and late apoptosis (37.02%), according to the results. A very tiny percentage of the characteristic apoptotic characteristics were also present in untreated cells.

In contrast to free β-carotene, we found that when the IC_50_ concentration of positively charged liposomal β-carotene was given to irradiated PC-3 cells at 5 grays, a significant increase in apoptosis was induced. This suggests that there is a connection between increased apoptosis and decreased positively charged liposomal β-carotene resistance. The expression of the apoptotic marker Annexin V was assessed in order to assess the impact of positively charged liposomal β-carotene on cell viability. After 48 h of exposure to positively charged liposomal β-carotene, the PC-3 cells showed greater levels of total apoptotic cell populations (Q2 + Q4) than the control cells, according to flow cytometric analysis. This suggests that the PC-3 cells were less able to withstand the impacts of positively charged liposomal β-carotene.

In addition to apoptosis, one significant strategy for controlling the growth of cancer cells is to stop the advancement of the cell cycle. Chemo preventive drugs can also induce cell cycle arrest, which may be a useful treatment for uncontrolled cell proliferation and survival in tumor cells. Thus, as potential therapies for reducing the growth of cancer cells, a large variety of naturally occurring substances that target cell cycle regulatory components have been studied [46].

**Fig. 18:**
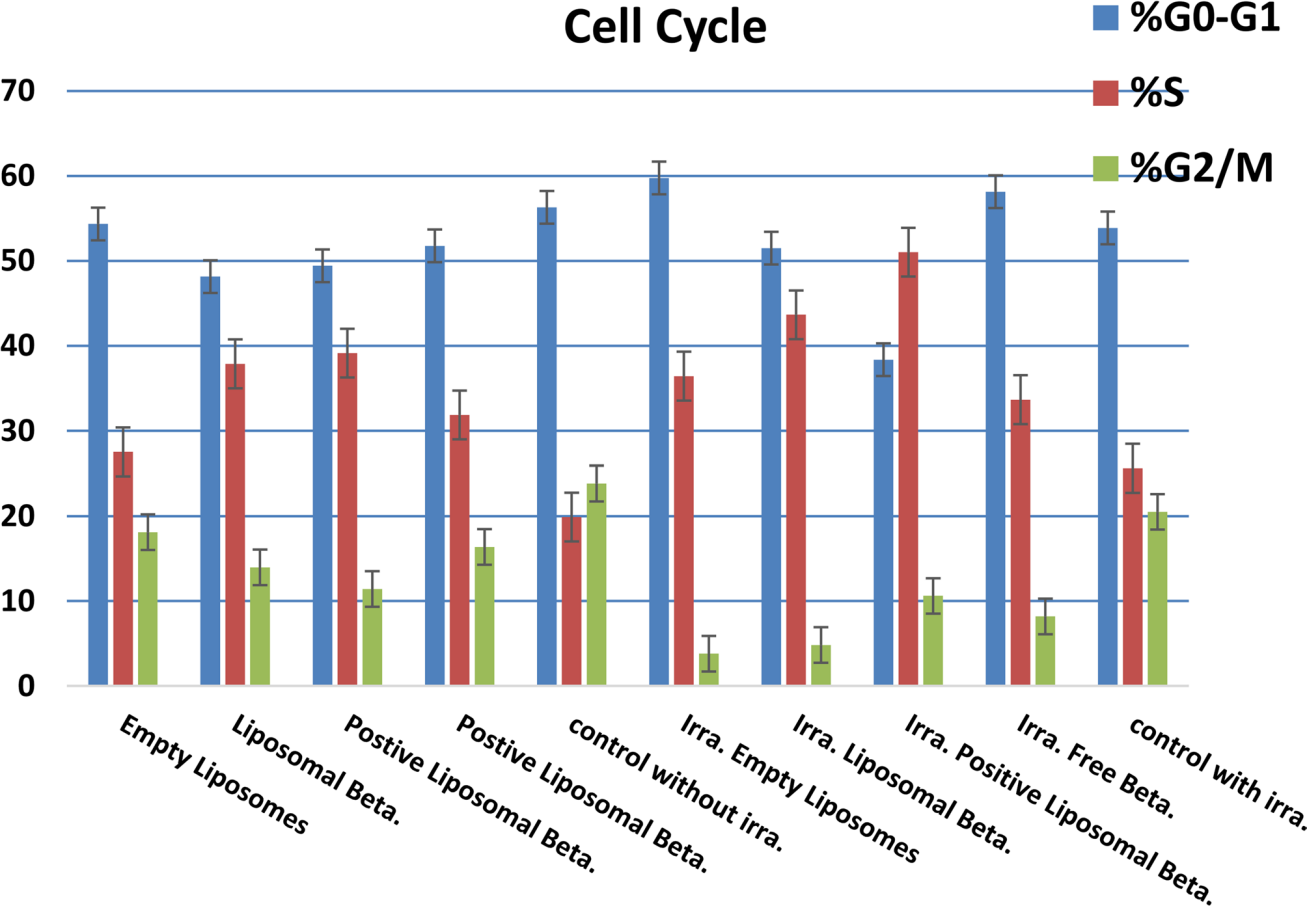
Cell cycle distribution of PC-3 cells analyzed by flow cytometry after 48 h incubation with IC₅₀ concentrations of free β-carotene, empty liposomes, liposomal β-carotene, and positively charged liposomal β-carotene, with and without irradiation. Data are presented as percentages of cells in different cell cycle phases compared to untreated controls (mean ± SD)

Apoptosis processes are usually accompanied by alterations in the cell cycle. The DNA content of the PC-3 cancer cell line during the cell cycle after treatment for 48 h with IC_50_ of free β-carotene, empty liposomes, liposomal β-carotene, and positively charged liposomal β-carotene was determined (Fig. 18).

The treatment with the IC_50_ of free β-carotene resulted in (51.77% and 31.87%) being followed by positively charged liposomal β-carotene (49.43% and 39.15%) cell accumulation in the G0-G1 and S phases, respectively, suggesting that free β-carotene followed by positively charged liposomal β-carotene inhibit prostate cancer cell growth by arresting cell cycling at the G1/S phase. Compared to the untreated control cells, 48.16% of PC-3 cells treated with liposomal β-carotene were arrested at the S phase, respectively (Fig. 18).

All treatments in this study resulted in the alteration of the cell cycle of PC-3 cells. The effect of liposomal β-carotene on PC-3 cells in this study causes cell cycle arrest in cancer cells at S phase, indicating that more cells were stopped at this stage. Positively charged liposomal β-carotene was reported to induce cell cycle arrest at the G1/S phase of prostate cancer cells, PC-3. This increased toxicity may be due to the preferential uptake of charged nanoparticles than that of the free drug. While β-carotene is mainly considered as a hydrophobic drug and could be entrapped in the hydrophobic core of the bilayer, it was observed closely adhered to the boundary surface within the liposomal assembly with external morphological alterations (Fig. 3B-C). This indicates that the liposomes may be physically hardened and associated with β-carotene at the surface disturbing the membrane packing property which manifested by slow leakage rate percentage of β-carotene which would lead to the higher cell cycle arrest at the G1/S phase of prostate cancer cells, PC-3.

The treatment of radiation-exposed PC-3 cells at 5 grays with the IC_50_ of positively charged liposomal β-carotene resulted in (58.14% and 33.67%) followed by liposomal β-carotene (51.52% and 43.66%) cell accumulation in the G0-G1 and S phases, respectively, suggesting that positively charged liposomal β-carotene followed by liposomal β-carotene inhibit radiation-exposed PC-3 cells growth by arresting cell cycling at the G1/S phase. Compared to the untreated control cells, 38.37% of radiation-exposed PC-3 cells treated with free β-carotene were arrested at the S phase, respectively (Fig. 18).

The carotenoid β-carotene selectively inhibits the development of breast cancer cells, according to [47]. An indication was observed on the more impact of free beta carotene or its Nano liposomal form in destroying breast cancer cells. Their results shed new light on a new treatment routine in which beta carotene or its liposomal form could be substituted with cyclophosphamide to raise its anticancer activity against MCF-7 cancer cell line.

### Limitations and future directions

Notable limitation of the present investigation is the comparatively low number of biological replicates utilized within the cytotoxicity, apoptosis, and cell cycle analyses. While a one-way ANOVA supported by post-hoc tests were performed, statistical power remained low. Consequently, differences found between formulation versions should be considered as biologically significant initial trends instead of statistical definitive results. Based upon the exploratory nature of present work, our initial goal has been to provide initial insights into prospective cytotoxic and radiosensitizing workings related to positively charged liposomal β-carotene. Thus, results here presented should be perceived as indicative of prospective possible biological effects, although additional extensive investigations with higher replicates will be needed to confirm their statistical soundness as well as their reproducibility.

Furthermore, this research was confined to in vitro experiments conducted with a single prostate cancer cell line and with a single dose of radiation and time of incubation. The stability, release profile, and possible off-target activity of liposomal β-carotene were not extensively explored, and comparisons against normal prostate cells were also not conducted.

Additional studies should consist of larger sample sizes with more replicates in order to make statistics more robust, advance studies to in vivo models, measure multiple irradiation regimens and time points, and examine molecular mechanisms of seen effects. Those steps will be of critical value in order to reveal therapeutic selectivity, safety, as well as potential combination of liposomal β-carotene with additional treatment modalities.

## Conclusion

The current study demonstrates that the administration of positively charged liposomal β-carotene in combination with 5 Gy ionizing radiation generates a synergistic cytotoxic effect against PC3 prostate cancer cells. The enhanced response is no doubt due to enhanced cellular uptake and membrane interaction mediated by the cationic liposomal formulation, as well as possible modulation of signaling pathways through radiation.

The results indicate that cationic liposomal β-carotene can be a radio sensitizer, allowing enhanced tumor control through possible reduced radiation exposure, consequently limiting off-target toxicity and enhancing therapeutic index.

Future research needs to outline the specific molecular pathways that this synergy results from i.e., through DNA damage, cell cycle arrest, and apoptosis, it as well as assess its effectiveness in the appropriate preclinical models. Exploration of combination with approved chemotherapeutics may also elucidate the promise of this Nano formulation in terms of augmenting cytotoxicity, bypassing drug resistance, and direct translation toward the clinician.

## Data Availability

The datasets used and/or analyzed during the current study are available from the corresponding author upon reasonable request.
